# PI31 is a positive regulator of 20S immunoproteasome assembly

**DOI:** 10.1242/jcs.263887

**Published:** 2025-05-23

**Authors:** Jason Wang, Abbey Kjellgren, George N. DeMartino

**Affiliations:** Department of Physiology, University of Texas Southwestern Medical Center, 5323 Harry Hines Boulevard, Dallas, TX 75390-9040, USA

**Keywords:** Proteasome, PI31, Immunoproteasome, Protein degradation, Proteasome regulator

## Abstract

Proteasome inhibitor of 31,000 Da (PI31) is a 20S proteasome-binding protein originally identified as an inhibitor of *in vitro* 20S proteasome activity. Although recent studies have elucidated a detailed structural basis for this inhibitory activity, the physiological significance of PI31-mediated proteasome inhibition remains uncertain, and multiple alternative cellular roles for PI31 have been described. Here, we report a role for PI31 as a positive regulator for assembly of the 20S immunoproteasome (20Si), a compositionally and functionally distinct isoform of the proteasome that is poorly inhibited by PI31. Genetic ablation of PI31 in mammalian cells had no effect on the cellular content or activity of constitutively expressed proteasomes but reduced the cellular content and activity of interferon-γ-induced immunoproteasomes. This selective effect results from impaired 20Si assembly, evidenced by the accumulation of 20Si assembly intermediates. Our results highlight a distinction in the assembly pathways of constitutive proteasomes and immunoproteasomes and indicate that PI31 plays a chaperone-like role in the selective assembly of 20S immunoproteasomes.

## Teaser

**Highlighted Article:** We show that PI31 knockout cells are defective in interferon-γ-induced biogenesis of 20S immunoproteasome (20Si) and suggest that PI31 functions as a 20Si assembly chaperone.

## INTRODUCTION

The proteasome is responsible for the degradation of most intracellular proteins in eukaryotes (Rock et al., 1994). Consequently, proteasome-dependent protein degradation mediates or regulates virtually every aspect of normal cellular physiology by controlling levels of proteins responsible for given processes. Despite the structural diversity of proteasome substrates and the breadth of different physiological states under which these proteins are degraded, proteasome function is highly selective and tightly regulated (Arkinson et al., 2025; Collins and Goldberg, 2017). This selectivity and regulation is exerted by multiple mechanisms, including regulated ubiquitylation of substrates that promotes selective proteasome-substrate interaction (Finley, 2009; Thrower et al., 2001), regulated post-translational modifications of the proteasome that directly affect its catalytic activity (Goldberg et al., 2021; Guo et al., 2017; VerPlank and Goldberg, 2017) and regulation of cellular proteasome content in response to changing physiological conditions (Altun et al., 2010; Cohen-Kaplan et al., 2016). An under-appreciated mode of proteasome regulation is the structural diversity of the proteasome itself (DeMartino and Gillette, 2007). The proteasome is a modular protease system consisting of several variants of a protease complex (20S proteasome or core particle) and multiple regulatory proteins to which they bind (e.g. 19S/PA700, PA28αβ, PA28γ, PA200, p97 and PI31) (Barthelme et al., 2014; DeMartino and Slaughter, 1999; Glickman and Raveh, 2005; Gray et al., 1994; Shibatani et al., 2006; Stadtmueller and Hill, 2011; Ustrell et al., 2002). The resulting holoenzymes feature distinct functional and regulatory properties imparted by their unique compositions. For example, the 20S-19S/PA700 holoenzyme (commonly referred to as the 26S proteasome) degrades ubiquitylated proteins by a mechanism linked to ATP hydrolysis (Arkinson et al., 2025; Collins and Goldberg, 2017). In contrast, 20S-PA28 complexes degrade certain non-ubiquitylated proteins in an ATP-independent fashion (Cascio, 2021; Mao et al., 2008). Additional structural and functional proteasome diversity is conferred by holoenzymes containing copies of two different regulators (Cascio et al., 2002; Hendil et al., 1998).

20S proteasomes are the common element of all proteasome holoenzymes. The 20S proteasome is a cylinder-shaped particle composed of four axially stacked hetero-heptameric rings (Baumeister et al., 1998; Bochtler et al., 1999). Each of the two identical outer rings is composed of seven different but homologous α-type subunits, while each of the two identical inner rings is composed of seven different but homologous β-type subunits (Baumeister et al., 1998; Groll et al., 1997). Three of the seven β subunits (β1c, β2c and β5c) feature N-terminal threonine residues that function as catalytic nucleophiles with varying specificities for peptide bond hydrolysis (cleavage after acidic, basic or neutral, and small hydrophobic residues, respectively) (Huber et al., 2016). The 20S proteasome exists in multiple isoforms characterized by unique complements of genetically distinct catalytic β subunits. For example, cells specialized for immune function constitutively express three so-called βi subunits (β1i, β2i and β5i), two of which (β1i and β5i) feature different cleavage specificities than their constitutive counterparts (cleavage after small hydrophobic and bulky hydrophobic residues, respectively) (Boes et al., 1994; Frentzel et al., 1993; Gaczynska et al., 1993; Huber et al., 2012; Tanaka, 2009). βi subunits are also conditionally expressed in cells exposed to certain cytokines, such as interferon-γ (IFNγ). These interferon-induced subunits are selectively incorporated into newly synthesized proteasomes in favor of their constitutively expressed β1c, β2c and β5c counterparts (Gaczynska et al., 1993; Khan et al., 2001). A specialized 20S proteasome composed of β1i/β2i subunits and β5t, a unique β5 subunit, is expressed in thymocytes (Murata et al., 2007, 2008). Other 20S proteasomes appear to have mixed populations of βc and βi catalytic subunits, although the basis for their formation is unclear (Dahlmann et al., 2000). Different 20S isoforms have different physiological functions. For example, the catalytic specificities of immunoproteasomes promote the production of antigenic peptides required for immune surveillance (Kincaid et al., 2012), and might be required for the clearance of oxidatively damaged proteins (Basler and Groettrup, 2021; Seifert et al., 2010). Likewise, the β5t subunit of thymo-proteasomes promotes the positive selection of CD8^+^ T cells (Murata et al., 2007, 2008). Thus, different 20S isoforms significantly expand the functional complexity of the proteasome system.

The 20Sc proteasome is assembled from its 28 constituent subunits by a highly ordered, stepwise process that is facilitated by five dedicated chaperones, as well as by chaperone-like functions of N-terminal pro-peptides of catalytic β subunits (Adolf et al., 2024; Budenholzer et al., 2017; Schnell et al., 2022; Zhang et al., 2024). A current model of 20S biogenesis, derived from extensive genetic, biochemical and structural data, indicates that the process begins with the formation of a complete α1–α7 subunit ring. This process is mediated by two heterodimeric chaperones, PAC1/PAC2 and PAC3/PAC4 (PAC1–4 are encoded by *PSMG1–4*, respectively) (Pba1/Pba2 and Pba3/Pba4, respectively in yeast) that remain bound to opposite faces of the completed α-subunit ring (Hirano et al., 2005). The chaperone POMP (Ump1 in yeast) then binds to the PAC3/PAC4 side of the α ring and initiates β-ring assembly by recruiting the pro-β2c subunit. The pro-peptide of β2c aids recruitment of the β3 subunit, which displaces PAC3/PAC4 and ejects it from the complex (Adolf et al., 2024). The remaining β subunits are then added in rank order (β4, β5, β6, β1 and β7) in a process directed by POMP and the pro-peptide of β5c. The resulting complex, often referred to as ‘15S’, consists of a half-proteasome (i.e. one α-subunit ring and one β-subunit ring) featuring bound PAC1/PAC2 and POMP chaperones and catalytic β subunits with unprocessed pro-peptides. Two half-proteasome complexes then join along abutting β-subunit rings to form the pre-holo-20S proteasome that initially retains all components of the individual 15S complexes. The fusion of two half-proteasomes induces a series of conformational changes that promotes the processing of β-subunit pro-peptides, degradation of POMP and dissociation of PAC1/PAC2 to generate the mature 20S proteasome (Adolf et al., 2024).

Proteasome inhibitor of 31,000 Da (PI31) was originally identified as an *in vitro* inhibitor of 20S proteasome activity (Ma et al., 1992; McCutchen-Maloney et al., 2000). Several recent reports have defined the detailed structural basis for this function; the natively unstructured C-terminus of PI31 enters the central catalytic chamber of the proteasome and directly interacts with the catalytic threonine residue of each β subunit (Hsu et al., 2023; Jespersen et al., 2022; Rawson et al., 2022). These interactions occur in a manner predicted to block hydrolytic activity of each catalytic site and are oriented in a manner that renders PI31 itself resistant to hydrolysis. The inhibitory features of PI31 have been exploited to develop small-molecule, subunit-specific proteasome inhibitors based on the structure of PI31 (Velez et al., 2023). Despite these *in vitro* biochemical findings, there is little evidence that PI31 functions as a global proteasome inhibitor in intact cells (Li et al., 2014). In contrast, PI31 has been proposed to regulate a variety of cellular processes with no apparent relationship to one another or to mechanisms involving direct inhibition of the proteasome's hydrolytic activity (Bader et al., 2011; Cho-Park and Steller, 2013; Kuo et al., 2015; Liu et al., 2019; Minis et al., 2019; Rathje et al., 2019; Zaiss et al., 2002).

Our initial characterization of PI31 as a proteasome inhibitor and our subsequent cryo-electron microscopy structure of the 20S-PI31 complex were performed using 20S proteasomes harboring constitutive catalytic β subunits (20Sc) (Hsu et al., 2023; Ma et al., 1992). We recently compared the effects of PI31 on purified 20Sc and 20S immunoproteasomes (20Si) *in vitro* and showed that PI31 had significantly attenuated inhibitory activity against 20Si (Wang et al., 2024). Surprisingly, this diminished inhibitory activity was largely accounted for by 20Si-catalyzed cleavage of PI31 at residues normally involved in proteasome inhibition. These findings indicate that PI31 interacts differently with the β subunits of 20Sc and 20Si and raise the possibility that that PI31 has different cellular roles in constitutive proteasomes and immunoproteasomes. Here, we describe a role for PI31 in the assembly of 20Si induced by IFNγ. Our data indicate that PI31 is an important factor that drives the assembly and maturation of IFNγ-induced 20S immunoproteasome.

## RESULTS

### PI31 knockout cells have normal proteasome content and activity under standard culture conditions

**Fig. 1.**
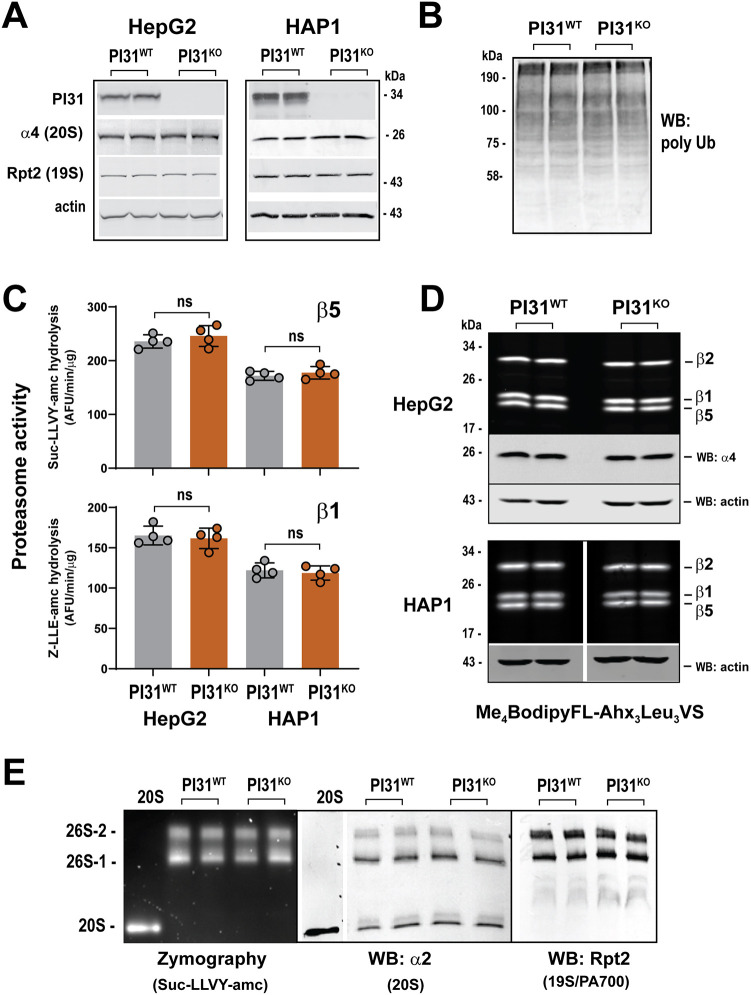
**PI31 knockout cells have normal levels and activity of constitutive proteasomes.** PI31 wild-type (WT) and knockout (KO) HAP1 and HepG2 cells were assessed for various features of proteasome content and activity. In all cases, extracts from WT and KO cells compare equal levels of total extract protein. (A) Extracts of HAP1 and HepG2 PI31 WT and PI31 KO cells were subjected to SDS-PAGE and western blotting for the indicated proteins. Duplicate lanes are samples from independent biological preparations. (B) Extracts of PI31 WT and PI31 KO HAP1 cells were subjected to SDS-PAGE and western blotting for polyubiquitylated proteins. Duplicate lanes represent independent biological preparations. (C) Proteasome activity was measured using the indicated substrates for extracts of PI31 WT and PI31 KO HAP1 and HepG2 cells. Each individual data point represents an independent biological experiment and is a mean value from triplicate assays. Bars represent mean values±s.d. of independent biological experiments and were analyzed by one-way ANOVA (ns, not significant; *P*>0.05). (D) Extracts of PI31 WT and PI31 KO HAP1 and HepG2 cells were treated with Me_4_BodipyFL-Ahx_3_Leu_3_VS and analyzed as described in the Materials and Methods. Indicated samples were subjected to western blotting for 20S α4 subunit and actin. Duplicate lanes are samples from independent biological experiments. Samples shown for HAP1 cell extracts are from the same gel, but intervening lanes are removed (see Fig. S4 for non-spliced image). (E) Extracts of PI31 WT and PI31 KO HAP1 cells were subjected to native PAGE and analyzed by zymography using Suc-LLVY-amc substrate, or by western blotting for the indicated proteins. ‘20S’ indicates purified 20S proteasome. Duplicate lanes are samples from independent biological experiments. The lane showing purified 20S proteasome was from the same gel and membrane as extract samples but was spliced from another part of membrane. WB, western blotting.

To investigate the physiological roles of the proteasome regulator, PI31, we ablated PI31 expression in HAP1 and HepG2 cells using CRISPR/Cas9 methodology. Disruption of the PI31-encoding gene (*PSMF1*) was confirmed by sequencing (Fig. S1), and no PI31 protein was detectable by western blotting in either PI31 knockout (KO) cell line (Fig. 1A). PI31 KO cells grew at indistinguishable rates from their parental counterparts under standard cell culture conditions replete with serum and nutrients (Fig. S2). Under these same conditions, the absence of PI31 had no appreciable effect on several measures of proteasome function, including steady-state levels of polyubiquitylated cellular proteins, rates of hydrolysis of model peptide substrates and labeling of the catalytic β subunits with the proteasome activity probe Me_4_BodipyFL-Ahx_3_Leu_3_VS (Fig. 1B–D). PI31 wild-type (WT) and KO cells had similar levels of representative proteasome subunits (Fig. 1A) and similar levels, complements and activities of intact proteasome complexes, as judged by native polyacrylamide gel electrophoresis (PAGE) coupled with zymography (for activity) or western blotting (for protein levels) (Fig. 1E). These results with PI31 KO cells are in accord with those of previous studies showing that depletion of PI31 content by RNA interference (RNAi) had no appreciable effect on global cellular proteasome content or activity under standard cell culture conditions (Li et al., 2014).

### PI31 KO cells have attenuated increases in immunoproteasome content and activity in response to IFNγ treatment

**Fig. 2.**
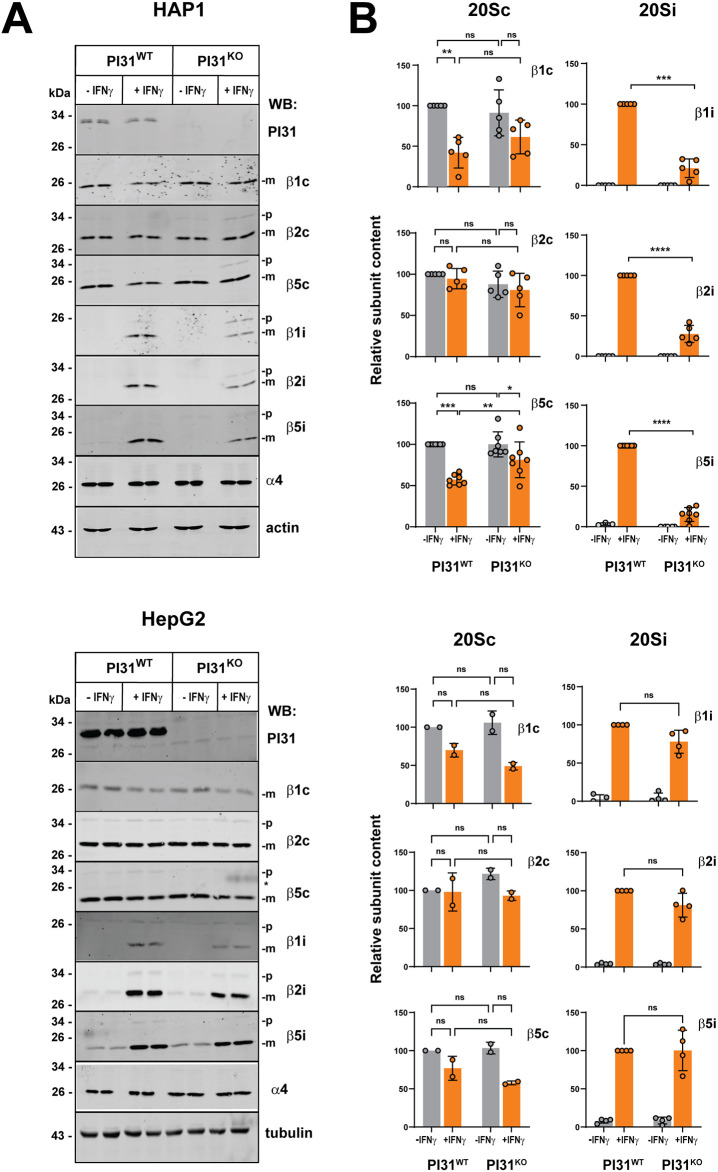
**PI31 KO cells have attenuated activities of immunoproteasome holoenzymes upon treatment with interferon-γ.** PI31 WT and KO HAP1 (top panels) and HepG2 (bottom panels) cells were exposed to 100 U/ml human interferon-γ (IFNγ) for 24 h. (A) Cell extracts from treated and untreated cells were normalized for total protein and subjected to SDS-PAGE and western blotting for the indicated proteins. Duplicate lanes are samples from independent biological experiments. Bands denoted ‘p’ and ‘m’ represent the pro-peptide and mature forms of β subunits, respectively. (B) Western blots of the indicated 20Sc and 20Si subunits were quantified using ImageStudio (LICOR) software from individual biological experiments, represented by data points; bars represent mean values±s.d. of independent biological experiments (HAP1 cells: b1c, b2c, b2i, *n*=5 each; b5c, b5i, *n*=7 each. HepG2 cells: b1c, b2c, b5c, *n*=7 each; b1i, b2i, b5i, *n*=4 each). Left column (20Sc): subunit levels of untreated PI31 WT cells were assigned a value of 100; subunit levels for other conditions are expressed relative to that value. Right column (20Si): subunit levels of IFNγ-treated PI31 WT cells were assigned a value of 100; subunit levels for other conditions are expressed relative to that value. Differences were analyzed by repeated measures two-way ANOVA and Tukey's HSD post-hoc test (ns, not significant; *P<0.05; **P<0.01; ***P<0.001; ****P<0.0001).

HAP1and HepG2 cells normally express β1c, β2c and β5c isoforms of catalytic proteasome subunits almost exclusively under standard culture conditions and therefore contain predominately constitutive forms of functional proteasome complexes. These cells, however, upregulate transcription of genes for β1i, β2i and β5i subunits when exposed to IFNγ. The IFNγ-induced βi catalytic subunits are selectively incorporated into newly synthesized proteasomes, commonly termed ‘immunoproteasomes’ (20Si), in lieu of their respective βc counterparts. Because we recently showed that purified PI31 interacts differently with purified 20Sc and 20Si proteasomes *in vitro* (Wang et al., 2024), we sought to determine whether PI31 exerts different effects on constitutive proteasomes and immunoproteasomes in intact cells. Accordingly, we treated PI31 WT and KO HAP1 and HepG2 cells with IFNγ and then compared their constitutive proteasome and immunoproteasome content and activity. In the absence of IFNγ, the levels of catalytic βc subunits were indistinguishable between PI31 WT and KO cells (Fig. 2A). These results are consistent with other comparisons of proteasome content and activity between PI31 WT and KO cells (Fig. 1). IFNγ had no general effect on the levels of βc subunits in either PI31 WT or KO cells, although protein levels declined significantly in a few instances (e.g. β1c and β5c in HAP1 WT cells; Fig. 2). These reductions in βc protein levels are likely to be a consequence of subunit turnover, and the distinctions in these effects might reflect different clearance rates of individual unassembled βc subunits within and between cells during the IFNγ treatment period.

As expected, IFNγ treatment for as little as 24 h greatly increased the expression of each βi subunit in both PI31 WT HAP1 and HepG2 cells (Fig. 2). IFNγ also increased βi content in PI31 KO cells, but the degree of increase was attenuated compared to that in WT cells. Attenuation was especially prominent in HAP1 cells, in which it was observed for each βi subunit and accompanied by an accumulation of unprocessed, pro-enzyme forms (Fig. 2). PI31 KO HepG2 cells also featured a trend for decreased IFNγ-induced βi subunit content, but this decrease was not statistically significant (Fig. 2B). Despite this distinction between HAP1 and HepG2 cells, further analysis described below shows that these PI31 KO cells share similar defects in the assembly of βi subunits into mature immunoproteasomes.

**Fig. 3.**
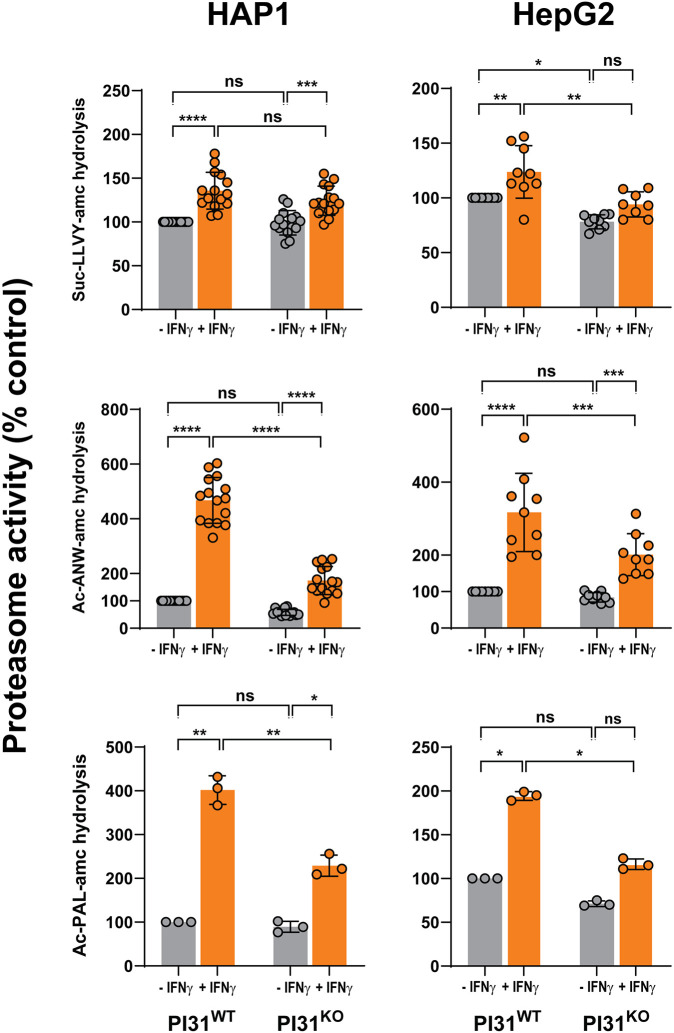
**PI31 KO cells have reduced IFNγ-induced immunoproteasome activity.** PI31 WT and PI31 KO HAP1 and HepG2 cells were treated with 100 U/ml human IFNγ for 24 h. Cell extracts were normalized for total protein and assayed for proteasome activity using Suc-LLVY-amc, Ac-ANW-amc and Ac-PAL-amc substrates, as described in the Materials and Methods. Mean activity values for extracts from untreated WT cells were measured and assigned a value of 100; activities of other conditions are expressed relative to that value. Activities of individual biological preparations are mean values of triplicate assays. Bars represent mean values±s.d. of independent biological experiments. Differences were analyzed by repeated measures two-way ANOVA and Tukey's HSD post-hoc test (ns, not significant; *P<0.05; **P<0.01; ***P<0.001; ****P<0.0001).

To determine the functional consequences of the lack of PI31 on content and activity of intact immunoproteasomes, we first measured and compared proteasome activity in extracts of PI31 WT and KO cells that had or had not been treated with IFNγ. IFNγ treatment had little or no effect on rates of hydrolysis of Suc-LLVY-amc, a substrate cleaved by both the β5c and β5i subunits of respective constitutive proteasomes and immunoproteasomes, in either PI31 WT or KO cells (Fig. 3; Fig. S3). Statistically significant increases were noted in some instances, but the magnitude of these effects was modest. In contrast, IFNγ treatment of PI31 WT cells promoted a large increase (2- to 6-fold) in the hydrolysis of Ac-ANW-amc and Ac-PAL-amc, peptide substrates cleaved with high specificity by the β5i and β1i subunits, respectively, of immunoproteasomes (Fig. 3; Fig. S3). These IFNγ-induced immunoproteasome activities, however, were significantly attenuated (40–60% reductions) in PI31 KO HAP1 and HepG2 cells (Fig. 3). A similar reduction in IFNγ-induced β2i activity was documented using the proteasome activity probe Me_4_BodipyFL-Ahx_3_Leu_3_VS. Although this activity probe labels both βc and βi subunits, the distinct migration of β2i on sodium dodecyl sulfate (SDS) gels permits its selective detection in samples containing both constitutive proteasomes and immunoproteasomes (Fig. S4). Thus, genetic ablation of PI31 expression reduced IFNγ-induced immunoproteasome activity in both HAP1 and HepG2 cells, despite its dissimilar effect on their overall levels of IFNγ-induced βi subunit content. This result suggests that the subunit content, per se, is not an accurate reflection of levels of intact, functional proteasome complexes. To confirm the role of PI31 in IFNγ-induced immunoproteasome activity, we employed RNAi as an independent method of reducing cellular PI31 content. HepG2 cells were transfected with siRNAs targeting PI31 prior to treatment with IFNγ. RNAi reduced PI31 content to undetectable levels (Fig. S5A). Consistent with results in PI31 KO cells and with previously reported results with HEK293 cells, RNAi of PI31 had little effect on the content or activity of constitutive proteasomes in the absence of IFNγ (Li et al., 2014). In contrast, IFNγ-induced immunoproteasome activity was significantly reduced in PI31 knockdown cells (Fig. S5B). This effect was accompanied by a reduction in the content of βi subunits (Fig. S5A). These results show that orthogonal methods of reducing cellular PI31 levels produce similar effects of inhibition of immunoproteasome activity.

**Fig. 4.**
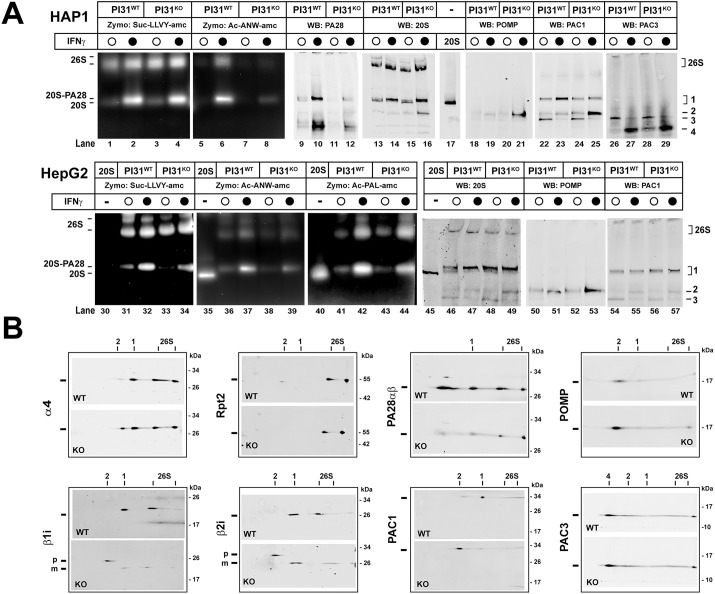
**PI31 KO cells have altered content and activity of IFNγ-induced proteasome complexes.** PI31 WT and PI31 KO HAP1 and HepG2 cells were exposed to 100 U/ml human IFNγ (‘•’) or control buffer (‘○’) for 24 h. (A) Cell extracts were normalized for total protein and subjected to native PAGE for zymography with the indicated proteasome substrates or western blotting for the indicated proteins. Purified 20S proteasome was electrophoresed as a standard. The lane showing purified 20S proteasome was from the same gel and membrane as extract samples but was spliced from another part of membrane. Complexes denoted 26S and 1–4 are described in the text. (B) Extracts of PI31 WT and KO HAP1 cells treated with IFNγ and subjected to native PAGE as in A, followed by second-dimension SDS-PAGE and western blotting for the indicated proteins. Positions denoted 26S, 1 and 2 correspond to those of the first-dimension native gel. ‘p’ and ‘m’ indicate positions of the unprocessed pro-peptide and mature forms of the indicated βi subunits. Similar results were obtained in four independent experiments.

To gain deeper insight into the basis of the diminished IFNγ-induced immunoproteasome activity in PI31 KO cells, we subjected extracts of IFNγ-treated and IFNγ-untreated PI31 WT and KO cells to native PAGE and analyzed the gels by zymography (for proteasome activity) and western blotting (for the content and composition of proteasome complexes). Most zymographic activity in untreated WT and KO cells was accounted for by constitutive 26S proteasomes (Fig. 4A, lanes 1, 3, 31, 33). Consistent with analysis of activities measured in whole-cell extracts, zymography of IFNγ-treated WT cells displayed substantially increased activities against immunoproteasome-selective substrates such as Ac-ANW-amc (selective for the β5i subunit; Fig. 4A, compare lanes 5 and 6, and lanes 36 and 37) and Ac-PAL-amc (selective for the β1i subunit; Fig. 4A, compare lanes 41 and 42; Fig. S5A). These immunoproteasome activities were accounted for by both 26S proteasomes and a band (Band 1) that migrated slightly slower than that for pure 20S proteasome (Fig. 4A; compare lanes 35 and 36–37, and lanes 40 and 41–41; Fig. S6A). Western blotting with an antibody that recognizes multiple α and β subunits of 20S proteasome revealed that the area of the gel near Band 1 activity contains several closely migrating bands (Fig. 4A, lanes 13–17 and lanes 45–49) that likely represent a mixture of immature pre-holo-20S, catalytically latent mature 20S and 20S-PA28αβ (both singly and doubly capped) holoenzyme complexes. Identification of these proteasome forms as components of Band 1 is supported by western blotting with antibodies against PA28αβ and PAC1 (Fig. 4A, lanes 9–12, 22–25 and 54–57; Fig. S6B). Additional evidence for the designations of these complexes is presented below. Notably, the activity of the 20S-PA28αβ complex was increased by IFNγ to a proportionally greater extent than that of 26S proteasomes and therefore appears to account for most of the IFNγ-induced increase in immunoproteasome activity. A selective increase in 20Si-PA28 activity in response to IFNγ treatment has been reported previously (Bose et al., 2001). Although the expected increase in PA28αβ expression by IFNγ observed in these experiments might play a role in the preferential increase of 20Si-PA28αβ content and activity, PA28αβ levels do not appear to limit formation of this holoenzyme because PA28 unassociated with the proteasome was observed in both treated and untreated cells (Fig. 4A, lanes 9 and 10; Fig. S6B). Nevertheless, IFNγ-induced zymographic immunoproteasome activities were markedly attenuated in both PI31 KO HAP1 and HepG2 cells (Fig. 4A; Fig. S6A). These results are consistent with the attenuated IFNγ-induced immunoproteasome activity of PI31 KO cells measured in whole-cell extracts and suggest that PI31 is required for normal induction of immunoproteasome activity in response to IFNγ (Fig. 4A, compare lanes 6 and 8, 37 and 39, 42 and 44). PI31 levels were not affected by IFNγ treatment, indicating that the basal content of PI31 was sufficient to support this proposed role (Fig. S7A).

In contrast to zymography, which allowed identification of immunoproteasome-selective activity, we were unable to directly distinguish constitutive proteasome and immunoproteasome content on native gels because immunoproteasome-specific antibodies available to us reacted poorly with intact native complexes. To circumvent this technical limitation, we subjected proteasome complexes resolved on native gels to second dimension SDS-PAGE and western blotting (Fig. 4B). This method confirmed identities assigned to these complexes on native gels and specifically identified immunoproteasomes. For example, Rpt2, a subunit of the 19S/PA700 regulatory complex common to both constitutive proteasome and immuno-26S proteasomes, was identified at horizontal positions of the SDS gel that aligned with complexes identified as 26S proteasomes on native gels. Likewise, α4, a subunit common to constitutive proteasomes and immuno-20S and -26S proteasomes, was detected at horizontal positions of the SDS gel that aligned with native gel bands assigned those identities. Consistent with the analysis of native gels alone, PA28αβ and 20S were detected at coincident horizontal positions on SDS gels that, in turn, aligned with the IFNγ-induced activity band from the native gel. This finding supports the conclusion that the activity of Band 1 is accounted for by a 20S-PA28 holoenzyme (Fig. 4A). PA28 was also identified in the regions corresponding to 26S proteasomes, indicating the presence of 20S-19S/PA700-PA28 hybrid complexes in these bands of zymographic activity. PA28 detected in the position characteristic of a proteasome-unbound form suggests that PA28 content did not limit formation of PA28-containing holoenzymes. Most importantly, mature βi subunits (denoted ‘m’) were identified in horizontal positions that aligned with those of both 20S/20S-PA28 (Band 1) and 26S proteasome complexes (Fig. 4B). Notably, the βi content in these bands was lower in IFNγ-treated PI31 KO cells than in corresponding WT cells. Collectively, these results support the conclusion that the reduced IFNγ-stimulated immunoproteasome activity of PI31 KO cells is a consequence of decreased immunoproteasome content.

### Cells lacking PI31 accumulate 20S immunoproteasome assembly intermediates containing 20S assembly chaperones and unprocessed βi subunits in response to IFNγ

In addition to detecting fully assembled, catalytically competent proteasomes, native PAGE also can identify intermediate complexes of the 20S assembly process. The native PAGE analysis of proteasome complexes described above revealed several bands that reacted with antibodies against 20S proteasome but migrated more rapidly than the purified 20S proteasome standard. We reasoned that these bands likely represent such 20S assembly intermediates (Fig. 4A, bands denoted 2, 3 and 4). For example, one of these bands (denoted Band 3; Fig. 4A, lanes 22, 24, 26 and 28) featured both PAC1 (a component of the PAC1/PAC2 heterodimeric chaperone) and PAC3 (a component of the PAC3/PAC4 heterodimeric chaperone) and therefore likely represents an early-stage complex composed of an intact α-subunit ring. The decreased content of Band 3 and the appearance of a new PAC3-containing band (Band 4, lanes 23, 25, 27 and 29) upon IFNγ treatment might indicate the progression of early-stage immunoproteasome assembly. Notably, another of these bands (Band 2; Fig. 4A, lanes 16 and 49) accumulated in samples from IFNγ-treated PI31 KO HAP1 and HepG2 cells. This finding suggests that attenuated immunoproteasome content and activity of IFNγ-treated PI31 KO cells is a consequence of defective or retarded 20Si assembly. To test this possibility, we examined the composition of the putative assembly intermediate in greater detail. Western blotting of native polyacrylamide gels showed that, in addition to subunits of 20S proteasome, Band 2 was detected by antibodies against the established 20S assembly chaperones POMP and PAC1 (Fig. 4A, lanes 18–25 and 50–57), but lacked the 20S chaperone PAC3 (Fig. 4A, lanes 26–29). Levels of the POMP and PAC1 chaperones in Band 2 were highest in extracts from IFNγ-treated PI31 KO cells (Fig. 4A, lanes 21, 25 and 53). Western blotting of whole-cell extracts confirmed that POMP levels were ∼2-fold greater in IFNγ-treated PI31 KO cells than in untreated cells (Fig. S7B). The protein composition of Band 2 is characteristic of the 15S half-proteasome assembly complex. In addition to the POMP and PAC1/PAC2 chaperones, this late-stage 20S assembly intermediate is also known to contain unprocessed pro-peptide forms of catalytic β subunits. To further characterize Band 2 from native gels, we again analyzed second-dimension SDS gels by western blotting. Both POMP and PAC1 were detected at a horizontal position of the SDS gel that aligned with Band 2 on the native gel (Fig. 4B). In agreement with the relative intensities of the western blotting signals in native gels, each of these proteins was present at higher levels in IFNγ-treated PI31 KO cells than in IFNγ-treated PI31 WT cells. Unprocessed, pro-peptide forms of βi subunits (denoted ‘p’) also were detected in the POMP/PAC1-containing Band 2 after second-dimension SDS-PAGE (Fig. 4B). These unprocessed βi subunits featured the expected higher-molecular masses than their processed mature forms that were located at positions that aligned with the fully assembled 20S (Band 1) and 26S proteasomes on native gels (Fig. 4B). Collectively, these findings support the conclusion that an assembly intermediate of IFNγ-induced 20Si proteasomes accumulates in PI31 KO cells and that this intermediate is similar or identical to the 15S half-proteasome.

**Fig. 5.**
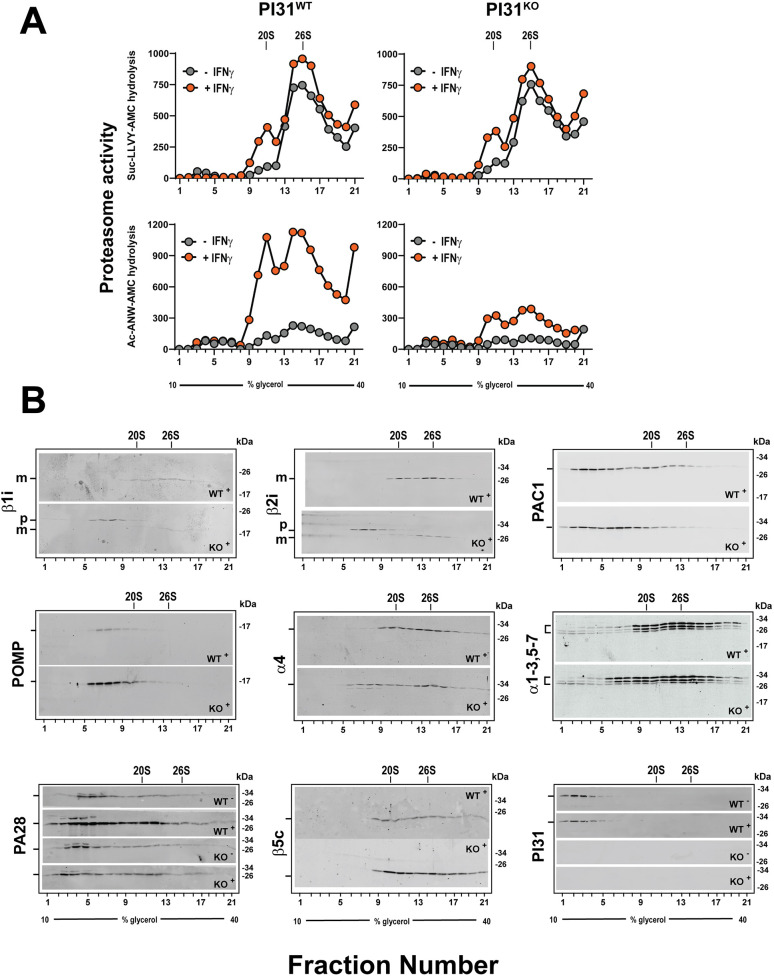
**PI31 KO cells have altered content and activity of IFNγ-induced proteasome complexes.** PI31 WT and PI31 KO HAP1 cells were exposed to 100 U/ml human IFNγ for 24 h. Cell extracts were normalized for total protein and subjected to 10–40% glycerol density gradient centrifugation. (A) Gradient fractions were assessed for proteasome activity with the indicated substrates. (B) Gradient fractions from A were subjected to western blotting for the indicated proteins. Blots show samples from treatments with (‘+’) or without (‘−’) IFNγ. 26S, 20S-PA28 and 20S show sedimentation positions of purified complexes as standards. ‘p’ and ‘m’ indicate unprocessed pro-peptide and mature forms, respectively, of the indicated βi subunits. Similar results were obtained in four independent experiments for activity measurements and in three independent experiments for western blotting.

Defective IFNγ-induced 20Si proteasome assembly was also demonstrated using glycerol density gradient centrifugation, an orthogonal method of identifying and comparing the content and activities of different proteasome complexes via functional assays and western blotting of gradient fractions (Fig. 5). This method confirmed key findings obtained by the native PAGE analysis described above. Thus, (1) untreated PI31 WT and KO cells had similar overall levels of constitutive proteasome activity, and this activity was accounted for mainly by constitutive 26S holoenzymes (Fig. 5A, top left and top right); (2) IFNγ greatly increased immunoproteasome activity in PI31 WT cells (Fig. 5A, bottom left); (3) IFNγ-stimulated immunoproteasome activity was accounted for by both 26S and 20S-PA28 complexes, but the latter complex was increased to a proportionally greater extent than the former (Fig. 5A, bottom left); and (4) IFNγ-induced immunoproteasome activity was markedly reduced in PI31 KO cells (Fig. 5A, bottom left and bottom right). Similar results were obtained with cells in which PI31 content was reduced by RNAi (Fig. S5C).

To further correlate these various proteasome activities with specific proteasome complexes, we subjected gradient fractions to western blotting (Fig. 5B). As expected, IFNγ-treated PI31 WT cells featured bimodal distribution profiles of representative 20S proteasome subunits, including α subunits and mature βc and βi subunits. The distribution profiles of these proteins were coincident with peaks of proteasome activity attributed to 26S (fractions 13–17) and 20S-PA28 (fractions 8–12) holoenzymes. The latter assignment was supported by a peak of PA28αβ that was coincident with both 20S subunits and proteasome activity. The level of PA28αβ in the latter peak was greater in IFNγ-treated than in untreated PI31 WT cells, a finding consistent with the conclusion that the 20Si-PA28 holoenzyme is preferentially formed in response to IFNγ treatment. The content and distribution of βi subunits in IFNγ-treated PI31 WT and KO cells were appreciably different. Thus, the level of mature βi subunits (denoted ‘m’) in peak fractions of 20S and 26S complexes was reduced in PI31 KO cells, and unprocessed pro-peptide forms of these subunits (denoted ‘p’) accumulated in slower-sedimenting fractions (Fig. 5B, fractions 6–8). These fractions also contained a peak of α subunits not present in IFNγ-treated wild-type cells, as well as enhanced peaks of POMP and PAC1. These findings are consistent with the accumulation of a complex similar to that identified as the 15S half-proteasome (Band 2) by the native PAGE analysis described above (Fig. 4). Collectively, these results are in excellent accord with those of the native PAGE analysis and support the conclusion that IFNγ-treated PI31 KO cells are defective in the assembly of 20Si proteasome.

### Knockdown of POMP reveals a distinct role for PI31 in immunoproteasome assembly

**Fig. 6.**
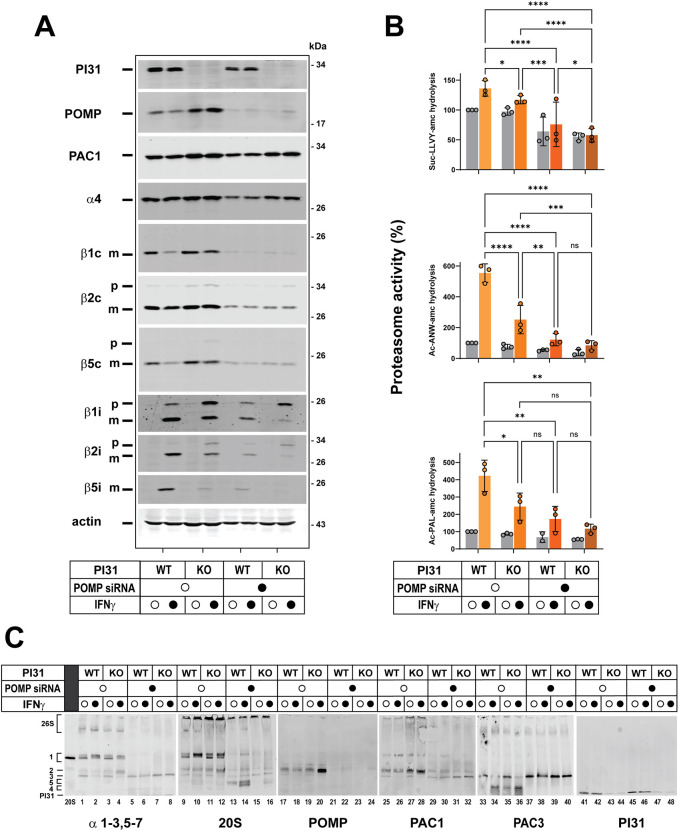
**Depletion of PI31 and POMP have distinct effects on IFNγ-induced proteasome activity and content.** PI31 WT and PI31 KO HAP1 cells were treated with POMP-coding or non-coding siRNAs for 24 h prior to treatment for 24 h with 100 U/ml human IFNγ or control medium. Cell extracts were normalized for total protein and subjected to the described analyses. (A) Extracts were subjected to SDS-PAGE and western blotting for the indicated proteins. (B) Extracts were assayed for proteasome activity against the indicated substrates. Mean activity values for extracts from untreated WT cells were assigned a value of 100; activities of other conditions are expressed relative to that value. Activities of individual biological preparations are mean values of triplicate assays. Bars represent mean values±s.d. of independent biological experiments. Differences were analyzed by repeated measures two-way ANOVA and Tukey's HSD post-hoc test (ns, not significant; *P<0.05; **P<0.01; ***P<0.001; ****P<0.0001). (C) Cell extracts were subjected to 3–8% native PAGE and western blotting for the indicated proteins. Bands denoted 26S and 1–5 are described in the text and correspond to those shown in Fig. 4. Similar results were obtained in six independent experiments for 20S, POMP and PI31, in three independent experiments for PAC1 and PAC3, and two independent experiments for α1–3,5–7.

The aberrant accumulation of a 20Si assembly intermediate in PI31 KO cells suggests that PI31 plays a normal role in 20Si assembly and maturation. To gain additional insight into this role, we compared the effects of PI31 KO on IFNγ-induced immunoproteasome content and activity with those resulting from depletion of POMP, an established 20S assembly chaperone. siRNAs directed against POMP decreased cellular POMP content to less than 5% of control levels in both IFNγ-treated and IFNγ-untreated PI31 WT and KO cells (Fig. 6A). Depletion of POMP inhibited IFNγ-induced immunoproteasome activities to a significantly greater extent than did KO of PI31 (>80% versus ∼50%, respectively), but depletion of POMP in PI31 KO cells had no significantly greater inhibitory effect than depletion of POMP alone (Fig. 6B). POMP knockdown decreased the steady-state levels of all βc and βi catalytic subunits in both IFNγ-treated and untreated PI31 WT cells (Fig. 6B). This result was mirrored by the effect of POMP knockdown on the hydrolysis of Suc-LLVY-amc, a measure of both constitutive proteasome and immunoproteasome activities. Moreover, the previously described decrease in IFNγ-induced βi subunit content in PI31 KO cells was more pronounced when these cells were also depleted of POMP (Fig. 6A).

To further examine the relative effects of POMP and PI31 on IFNγ-induced immunoproteasome assembly, extracts from the cellular conditions described above were electrophoresed on native gels and blotted with antibodies that detect various 20S proteasome subunits, POMP, PAC1 (a monitor of the PAC1/PAC2 heterodimer), PAC3 (a monitor of the PAC3/PAC4 heterodimer) and PI31. Consistent with the results from the activity and western blotting assays described above, depletion of POMP significantly reduced the content of intact proteasome holoenzymes in both PI31 WT and KO cells regardless of IFNγ treatment (Fig. 6C, lanes 5–8 and 13–16). This effect likely reflects a common role for POMP in the early-stage assembly of both constitutive proteasomes and immuno-20S proteasomes. As expected, the content of Band 2, the late-stage POMP-containing assembly intermediate that accumulates in IFNγ-treated PI31 KO cells, was also significantly reduced in POMP knockdown samples (Fig. 6C, lanes 21–24). In place of Band 2, POMP knockdown samples featured a prominent faster-migrating band (annotated Band 3) containing 20S α subunits, PAC1 and PAC3 (Fig. 6C, lanes 5–8, 29–32 and 37–40, respectively). The presence and composition of Band 3 in the absence of POMP indicate that Band 3 represents the α-subunit ring assembly intermediate that forms prior to the action of POMP and incorporation of β subunits. Accordingly, a PAC3-containing band that migrated more rapidly than Band 3 was detected in extracts from IFNγ-treated PI31 KO cells with normal POMP content (Fig. 6C, lanes 33–36). This finding is consistent with current models in which PAC3/PAC4 ejection from the α-ring-POMP-PAC1/PAC2 complex is coincident with initial insertion of βi subunits on the assembled α ring (Fig. 6C, lanes 33–36, annotated as Band 4). Because PI31 had no effect on the content of Band 3 in POMP-depleted cells, its role in immunoproteasome assembly is likely to be exerted a step after the PAC1/PAC2-PAC3/PAC4-mediated formation of the α-subunit ring. Such steps might include insertion of one or more βi subunits into the progressively growing assembly complex or the joining of two 15S half-proteasomes. Thus, the general loss of β subunit content in POMP knockdown cells and the enhanced loss of βi subunit content upon depletion of both POMP and PI31 could reflect the instability of these subunits when their insertion into assembly complexes is blocked. In fact, a band likely containing β subunits (annotated Band 5 in Fig. 6C, lanes 13 and 14) was present at much lower levels when POMP was depleted from PI31 KO cells than from PI31 WT cells (Fig. 6C, lanes 9–16; see Discussion). Unlike POMP, PAC1 and PAC3, we were unable to detect PI31 as a component of Band 2, Band 3 or any other identifiable assembly intermediate. PI31 migrated near the dye front, a position that was indistinguishable from that of isolated, purified PI31 (Fig. 6C, lanes 41–42 and 45–46). Similarly, PI31 was not detectably associated with assembly intermediates identified by glycerol density gradient centrifugation (Fig. 5). These findings suggest that any role of PI31 in the assembly of IFNγ-induced immunoproteasomes is characterized by highly transient interactions with intermediate complexes.

## DISCUSSION

**Fig. 7.**
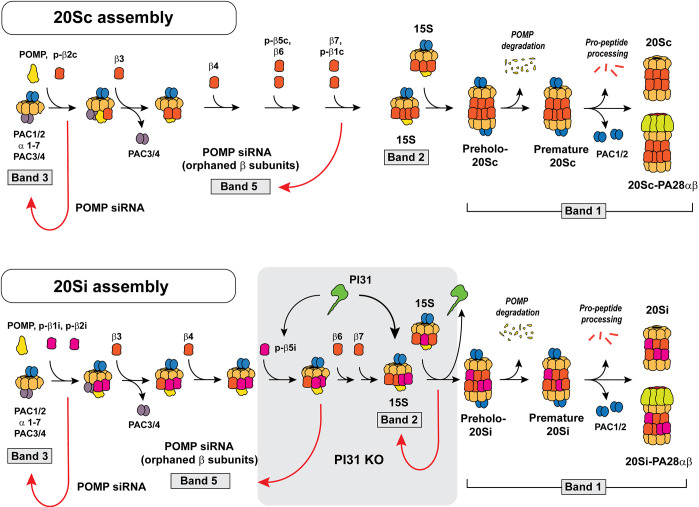
**Models of 20Sc and 20Si proteasome assembly.** Top: model of 20Sc assembly consistent with current literature. Bottom: model of IFNγ-induced 20Si assembly. Gray box indicates possible sites of PI31 action based on the present study. Denoted bands correspond to proteasome complexes identified on native gels (Figs 4 and 6) and discussed in the text. Red arrows indicate effects of PI31 KO on the indicated bands.

The formation of large multisubunit proteins often requires chaperones to coordinate the spatial and temporal complexities of the assembly process. The tightly orchestrated assembly of the 28-subunit eukaryotic 20S proteasome is aided by five dedicated chaperones: POMP and two heterodimeric proteins, PAC1/PAC2 and PAC3/PAC4 (Kunjappu and Hochstrasser, 2014; Matias et al., 2010; Murata et al., 2009; Schnell et al., 2022; Tomko and Hochstrasser, 2013). Moreover, N-terminal pro-peptides of several constituent catalytic β subunits contribute additional chaperone-like functions to 20S assembly (Schnell et al., 2022). Extensive genetic, biochemical and structural studies have provided a detailed and increasingly comprehensive understanding of the multistep pathway, including the mechanisms and relative roles of each protein, in the assembly of the 20S proteasome (Fig. 7).

Higher eukaryotes can express multiple 20S proteasome isoforms characterized by unique complements of genetically and functionally distinct catalytic β subunits. Although the assembly pathways of these different 20S isoforms are broadly similar, specific aspects of the process appear to differ. Such distinctions might be the basis for the selective formation of given isoforms under different physiological conditions. For example, the selective formation of immunoproteasomes upon IFNγ-induced expression of βi subunits seems to be driven by the initial preferential addition of β1i and β2i to the α-subunit ring in lieu of β2c (Bai et al., 2014). This process appears to divert subsequent steps of 20S assembly in favor of 20Si (Fig. 7).

The current work identifies PI31, a poorly understood proteasome-binding protein, as a positive regulator of 20Si assembly. Depletion of cellular PI31 by genetic KO or knockdown methodologies stalls IFNγ-induced 20Si assembly at an intermediate stage similar or identical to that featuring the well-characterized 15S half-proteasome assembly complex (Fig. 4, Band 2). This finding suggests that PI31 plays a role in a process required for late-stage insertion of one or more β subunits into the complex and/or for fusion of two fully assembled half-proteasomes to form the pre-holo-20Si complex (Fig. 7, gray-shaded box). The reduced content of mature 20Si proteasomes caused by the absence of PI31 limits the formation of functional 20Si-PA28 and 26Si proteasome holoenzymes. Notably, the majority of reduction in IFNγ-induced immunoproteasome activity in PI31 KO cells is accounted for by decreasing levels of 20Si-PA28 holoenzymes. Although the preferential formation of this holoenzyme in WT cells could be driven, in part, by the well-established IFNγ-increased expression of genes encoding PA28α and PA28β subunits (Ahn et al., 1995; Fig. S7C), PA28 levels do not appear to be a limiting factor for holoenzyme formation under any condition examined here. Moreover, the IFNγ-increased formation of 20Si-PA28αβ complexes was prominent in HepG2 cells in which PA28 expression was not noticeably enhanced by 24 h of treatment. It is unclear whether some aspect of IFNγ signaling actively promotes 20Si-PA28 formation or whether any factor other than lower 20Si levels contributes to reduced 20Si-PA28 levels in IFNγ-treated PI31 KO cells.

Despite the robust effect of PI31 KO on decreasing cellular immunoproteasome content and activity, neither the mechanism of this effect nor the basis for its apparent specificity for the immunoproteasome is clear. Unlike established 20S assembly chaperones (POMP, PAC1/2 and PAC3/4), PI31 was not detected as a component of any assembly complex resolved here. This might be a consequence of transient or weak interactions of PI31 with these complexes that preclude complex identification by native PAGE or density gradient centrifugation methodologies. Attempts to capture putative PI31-containing intermediates by immunoprecipitation methods were also unsuccessful. It is possible that selective degradation of PI31 by 20Si is part of the normal assembly process and contributes to our failure to detect PI31-containing assembly intermediates. 20Si-selective PI31 proteolysis has been documented *in vitro*, and degradation of POMP also occurs during normal 20S assembly (Han et al., 2025; Schnell et al., 2022; Wang et al., 2024). Regardless of the inability to detect a physical association of PI31 with assembly intermediates, several structural and functional properties of PI31 lead us to posit that PI31 normally functions as a chaperone for 20Si assembly via direct interactions with proteasome subunits. First, high-resolution cryo-electron microscopy structures of 20S-PI31 complexes reveal extensive interactions of PI31 with 20S proteasome subunits. These interactions include those between the carboxyl terminus of PI31 and each catalytic β subunit (Hsu et al., 2023; Jespersen et al., 2022; Rawson et al., 2022). Although these interactions could be unique to fully assembled 20S proteasomes, it is reasonable to assume that closely related interactions also occur between PI31 and proteasome assembly complexes or individual subunits. Such interactions could result in the recruitment or delivery of one or more β subunits to assembly complexes and/or in induction of conformational changes within assembly complexes required for progression of the assembly process. Interactions between PI31 and individual β subunits, as might be involved in delivery of a subunit to an assembly intermediate, would likely not be detected at the resolution of native PAGE or glycerol density gradient centrifugation. In such a case, the absence of PI31 might promote turnover of the unincorporated subunit that accounts for its diminished content by western blotting. Second, we recently identified differential interactions of PI31 with the catalytic β subunits of intact 20Sc and 20Si proteasomes (Wang et al., 2024). These differences provide a potential basis for the selectivity of PI31 on 20Si assembly. For example, specific features of PI31 binding to βi subunits might direct their selective incorporation into assembly complexes. Alternatively, preferential binding of PI31 to βc subunits might sequester βc subunits from productive association with assembly complexes, thereby favoring incorporation of βi subunits into newly synthesized proteasomes. Such an inhibitory effect of PI31 on formation of 20Sc, however, would likely demand additional forms of regulation to prevent interference of PI31 with normal assembly of 20Sc in the absence of IFNγ. Third, PI31 harbors an HbYX motif at its carboxyl terminus. This motif is found in other 20S regulatory proteins in which it supports holoenzyme stability and induces opening of the substrate access pore (Rabl et al., 2008; Smith et al., 2007). HbYX motifs, however, are also found in PAC1 and in yeast Pba1/Pba2 20S chaperones in which they play specific roles in 20S assembly (Schnell et al., 2022). Because the HbYX motif is not required for binding of PI31 to, or inhibition of, intact 20S proteasomes, its conservation in PI31 might indicate a role analogous to that of other assembly chaperones (Li et al., 2014). Fourth, endogenous PI31 levels are at least an order of magnitude lower than levels of 20S proteasomes in mammalian cells (Beck et al., 2011; Fabre et al., 2014; Kulak et al., 2014; Schwanhäusser et al., 2011). This feature is consistent with its possible role as an assembly chaperone and limits other possible physiological roles. Moreover, other established 20S assembly chaperones have cellular levels similar to those of PI31. Finally, we note a previous report that demonstrated inhibition of IFNγ-induced immunoproteasome activity after massive overexpression of PI31 (Zaiss et al., 2002). Similar to the current results with PI31 KO cells, this effect was accompanied by reduced assembly of immunoproteasomes and an accumulation of unprocessed pro-peptide forms of βi subunits in assembly intermediates (Zaiss et al., 2002). Although no mechanism for this effect was described, similar phenotypes are often caused by both depletion or overexpression of proteins that function as scaffolds and coordinate interactions among different proteins. We have also observed inhibition of IFNγ-induced proteasome activity upon massive PI31 overexpression in several cell lines (Fig. S8). This inhibition was not accompanied in our studies by a detectable accumulation of unprocessed βi subunits. Moreover, unlike PI31 KO, PI31 overexpression also inhibited constitutive proteasome activity in both IFNγ-treated and untreated cells (Fig. S8). Thus, the mechanism(s) by which PI31 overexpression inhibits proteasome activity under these conditions remains unclear. The inhibitory effects of PI31 overexpression on IFNγ-induced proteasome content and activity also complicated our attempts to rescue defects in PI31KO cells by exogenous expression of PI31 in these cells. Critical testing of the effect of such rescue experiments will require expression of PI31 at levels that match those of endogenous PI31 in WT cells.

Although we favor a model in which PI31 supports 20Si assembly via direct interactions with proteasome subunits or chaperones, we cannot exclude models based on other possible mechanisms of action. For example, PI31 is known to bind to FBXO7, a subunit of an SCF-type E3 ligase (Kirk et al., 2008). Thus, an FBXO7-PI31-containing ligase might selectively target a protein for which degradation is required for normal expression of βi subunits or for normal assembly of βi subunits into mature 20Si proteasomes. A role for PI31 in the transcription of βi subunits by this or other mechanisms appears to be excluded by our results showing uninhibited expression of IFNγ-induced βi subunit mRNAs in PI31 KO cells (Fig. S9). Indirect effects of PI31 on 20Si assembly, via either degradation-dependent or degradation-independent mechanisms, could also involve PI31-mediated regulation of a component or activity of post-transcriptional IFNγ signaling pathways possibly required for 20Si assembly. Finally, PI31 might be required to support enhanced rates of 20S proteasome assembly, such as could occur in response to IFNγ, rather than to promote selective assembly of 20Si. These speculations and other possible determinants of PI31 action on proteasome assembly will require additional investigation.

The current results add another function to the growing list of reported physiological roles for PI31. In addition to its original description as an *in vitro* inhibitor of purified 20S proteasome, PI31 has been described as a direct activator of the 26S proteasome (Bader et al., 2011), an assembly factor for 26S proteasomes (Cho-Park and Steller, 2013), a negative regulator of antigen presentation (Zaiss et al., 2002), an adaptor for dynein-mediated proteasome transport on neuronal axons (Liu et al., 2019), a negative regulator of viral replication (Kuo et al., 2015) and a regulator of mitochondrial function (Al Rawi et al., 2024). Although the relative physiological significance of these various functions remains to be determined, we propose that the unique structure of PI31 can enable it to participate in multiple and diverse processes, the given manifestations of which depend on specific cellular and physiological conditions.

## MATERIALS AND METHODS

### Generation of PI31 KO cell lines

The *PSMF1* gene (UniProt ID Q92530) encoding PI31 was disrupted in HAP1 and HepG2 cells with sgRNA 5′-CCTTGTGAAAGCCATCACCG-3′ and protospacer adjacent motif sequence TGG (GenScript) for HepG2 cells, and sgRNA 5′-CATCCTTATACTCATACCG-3′ for HAP1 cells, each targeting exon 2. Cells were plated into six-well plates and transfected with 5 µl Lipofectamine 3000 and 5 µl P3000 (Invitrogen) containing 1 µg pLentiCRISPR v2 plasmid. Twenty-four hours after transfection, cells were exposed to selection medium with 5 µg/l puromycin for an additional 48 h. The selection medium was removed, and monoclonal cells were screened by limiting dilution. Gene disruption was confirmed by Sanger sequencing and western blotting. Sequencing data demonstrated a 10 bp deletion for HAP1 cells and a 1 bp deletion for HepG2 cells in exon 2 of the *PSMF1* gene (Fig. S1).

### Cell culture

Human cell lines (HAP1, HepG2 and HeLa) were obtained from American Type Culture Collection. Cells were cultured in Iscove's modified Dulbecco's medium (HAP1) and Dulbecco's modified Eagle medium (HepG2 and HeLa) supplemented with glutamine, 10% fetal bovine serum and penicillin (50 IU/ml)/streptomycin (50 µg/ml), at 37°C with 5% CO_2_. Cells were routinely tested inspected for bacterial, fungal and mycobacteria contamination. The latter was accomplished by selective PCR or by commercially available test strips.

### IFNγ treatments

Cells were treated with 100 U/ml recombinant human IFNγ (Thermo Fisher Scientific) as indicated in specific experiments. Prior to treatments, cells were plated at densities of 1–1.5×10^6^ cells per 100 mm plate and exposed the following day for durations indicated in the figure legends.

### Preparation of cell extracts

Cell extracts were prepared for proteasome assays, gel electrophoresis and glycerol density gradient centrifugation. Cells were washed three times with ice-cold PBS. Washed cells were collected in hypotonic lysis buffer [20 mM Tris-HCl (pH 7.6 at 37°C), 20 mM NaCl, 1 mM DTT, 5 mM MgCl_2_, 100 µM ATP, 0.1% NP-40]. For analyses of proteasome assembly intermediates in experiments in which activity was not measured, 10 µM MG132 (Sigma-Aldrich, cat. no. 474790) was added to the lysis buffer. Cells were mechanically lysed by 10–15 passes through a 27.5 G needle, and supernatants were collected following centrifugation at 16,000 ***g*** for 20 min at 4°C. Samples subjected to native PAGE were centrifuged at 20,000 ***g*** for 20 min at 4°C. Protein content of cell lysates was determined using detergent-compatible BCA assays (Pierce BCA Protein Assay Kit, Thermo Fisher Scientific) as described by the manufacturer. Protein concentrations were determined using bovine serum albumin as a standard.

### Measurement of proteasome activity using fluorogenic peptide substrates

Proteasome activity was measured in cell extracts by monitoring the fluorescence of 7-amino-4-methylcourarin (amc) cleaved from amc-linked peptide substrates Suc-LLVY-amc, Ac-ANW-amc or Ac-PAL-amc. 10 µl of cell extract was added to 150 µl of 50 µM peptide substrate in an assay buffer consisting of 20 mM Tris-HCl, pH 7.6, 20 mM NaCl, 5 mM MgCl_2_ and 100 µg ATP. Assays were conducted in triplicate in 96-well plates using a BioTek Synergy plate reader at 37°C. Fluorescence (excitation, 380 nm; emission, 460 nm) was measured every 45 s for 21 min. Control assays contained lysis buffer without cell extract. Rates of production of amc fluorescence were determined by instrument software and expressed as arbitrary fluorescent units/min/µg extract proteins. All reported rates are from assays in which amc production was linear with respect to incubation time and with respect to extract concentration.

### Measurement of proteasome activity using proteasome activity probes

Proteasome activity was also measured using activity-based probe labeling of Me_4_BodipyFL-Ahx_3_Leu_3_VS for each active site. Whole-cell lysates were incubated in 1 µM activity-based probe at 37°C for 1 h. Reactions were terminated by the addition of 5× SDS sample buffer. Extracts were subjected to SDS-PAGE, transferred to nitrocellulose membranes and imaged on an Odyssey M imager (LICOR) at 520 nm.

### Western blotting

Cell extracts were denatured in SDS sample buffer under reducing conditions and denatured at 100°C for 5 min. Known amounts of protein were separated by SDS-PAGE (10–12% acrylamide) and transferred to 0.2 µm nitrocellulose membranes in transfer buffer containing 20% MeOH. Membranes were then blocked in 5% milk in TBST buffer (20 mM Tris-HCl, pH 7.5, 150 mM NaCl, 0.05% Tween 20) for 1 h at room temperature. Primary antibodies were incubated overnight at 4°C. Secondary antibodies with IRDye were obtained from LICOR and used at 1:10,000 dilution. Near-infrared emission from IRDye was imaged using Odyssey Dlx or Odyssey M (LICOR) at channel 700 or 800 nm depending on the secondary antibody. Original and uncropped images of western blots can be found in Fig. S10. In some instances, full uncropped images are not available because transfer membranes were cut into separate strips for probing with individual antibodies.

The following primary antibodies were used at 1:2000 to 1:500 dilutions: anti-20Sc β2 subunit (rabbit monoclonal IgG; Cell Signaling Technology, 13207); anti-20Sc β2i subunit (rabbit polyclonal; Cell Signaling Technology, 78385); anti-20S α4 subunit (mouse monoclonal IgG1, MCP34; Enzo, BML-PW8120); anti-20S α1–3/5–7 (mouse monoclonal IgG1, MCP231; Abcam, ab22674); anti-PI31 (rabbit polyclonal; ABclonal, BML-PW9710); anti-PAC1 (rabbit polyclonal; Cell Signaling Technology, 13378); anti-PAC3 (mouse IgG2b; Proteintech, 67466-1-Ig); anti-POMP (rabbit monoclonal IgG; Cell Signaling Technology, 15141); anti-actin (mouse monoclonal C4; Sigma-Aldrich, MAB1501); anti-β-tubulin (rabbit polyclonal; Cell Signaling Technology, 2146); anti-Flag (mouse monoclonal; Thermo Fisher Scientific, MA1-91878); anti-ubiquitin (mouse monoclonal (FK2); Sigma-Aldrich, ST1200); and anti-20S α2 subunit (mouse monoclonal IgG MCP21, a gift from Klaus Hendil, Department of Biology, University of Copenhagen, DK-2200 Copenhagen N, Denmark).

Polyclonal antibodies against human proteins generated in rabbits were prepared in the DeMartino laboratory against the indicated antigens: 20S β1 subunit (antigen peptide: N-LAAIAESGVERQVLLGDQIPKFAVATLPPA-C) (Wang et al., 2024); 20S, β5 subunit (antigen peptide: N-WIRVSSDNVADLHEKYSGSTP-C) (Wang et al., 2024); 20S, β1i subunit (antigen peptide: N-IYLVTITAAGVDHRVILGNELPKFYDE-C (Wang et al., 2024); 20S β5i subunit (antigen peptide: N-WVKVESTDVSDLLHQYREANQ-C (Wang et al., 2024); 20S E446 protein (antigen-purified 20S proteasome) (McGuire et al., 1988); PA28αβ protein (antigen-purified PA28αβ) (Ma et al., 1993); PA700 Rpt2 subunit (antigen peptide: N-ENVLYKKQEGTPEGLYL-C); and PA700 Rpt5 subunit (antigen peptide: N-ILEVQAKKKANLQYYA-C).

### Native PAGE

Native PAGE was conducted using 3–8% polyacrylamide Tris-acetate gels (Life Technologies) as described previously (Kim and DeMartino, 2011; Kim et al., 2013). In brief, samples were mixed with 5× native PAGE sample buffer, applied to the loading wells and electrophoresed for 2.5–3.0 h at 100 V at 4°C with a 90 mM Tris-borate running buffer containing 5 mM MgCl_2_ and 100 µM ATP. Following electrophoresis, proteasome activity of separated bands was performed by incubating gels in 50 µM fluorogenic peptide substrate (Suc-LLVY-amc, Ac-ANW-amc or Ac-PAL-amc) in the presence of 5 MgCl_2_ and 100 µM ATP. Incubations were conducted at 37°C for 30 min. Gels were imaged using a ChemiDoc UV transilluminator (Bio-Rad) using the fluorescein channel. For immunoblotting of native PAGE samples, gels were transferred and blotted under the same conditions described for western blotting above.

### Second-dimension SDS-PAGE

Second-dimension SDS-PAGE was conducted by excising individual lanes of native polyacrylamide gels. Excised gel lanes were incubated in 1× SDS sample buffer at room temperature for 10 min and adhered horizontally to the top of SDS 12% polyacrylamide gel with electrode buffer containing 0.5% agarose. After transfer, first-dimension native lane boundaries were visualized by Ponceau S staining and marked prior to blocking. SDS-PAGE and western blotting were conducted as described above.

### Glycerol density gradient centrifugation

Glycerol density gradient centrifugation was conducted as described previously (Koulich et al., 2008). Cell extracts (∼500 µg total extract protein) were applied to a 1.95 ml 10–40% linear glycerol gradient in a buffer containing 50 mM Tris-HCl (pH 7.6, 4°C), 100 µM ATP, 5 mM MgCl_2_, 1 mM DTT. Centrifugation was carried out for 3.5 h at 4°C in a Beckman Optima TL ultracentrifuge at 55,000 rpm in a TLS-55 rotor (Beckman-Coulter). 21 fractions (100 µl/fraction) were collected and analyzed as described for given experiments.

### RNAi

RNAi for knockdown of POMP and PI31 was conducted using ON-TARGETplus SMARTpool siRNA mixes (Horizon Discovery, L-016844-01-0005 and L-012320-01-0005, respectively). Pooled siRNA mixes contained four individual target sequences, which were also validated separately. Specific siRNAs used individually for POMP were GGGUCUAUUUGCUCCGCUA and UCAUGAUCUUCUUCGGAAA. Specific siRNAs for PI31 were CAACAUAUAUCCUCGACCA and GAGUGGAAAGAGCACGAUA. 25 nM siRNAs were transfected into cells using DharmaFECT1 according to the manufacturer's recommendations for six-well plate scale. A pool of control non-targeting siRNAs was used as a negative control (Horizon Discovery, D-001810-10-05). PI31 and POMP protein expression was assessed at 24 and 48 h to determine an appropriate time course. Following PI31 or POMP knockdown, cells were exposed to control or human IFNγ (100 U ml^−1^) for 24 h. Cells were harvested and assayed as described above.

### Overexpression of PI31

Overexpression of PI31 was performed as described previously (Hsu et al., 2023). Briefly, cells were transfected with Lipofectamine 3000/P3000 (Invitrogen) with a pCMV3-N-FLAG-PI31 construct (Sino Biological, HG17079-NF). 24 h after transfection, transfection medium was removed, and cells were exposed to IFNγ or control treatments for the amounts of time indicated in figure legends.

### Measurement of mRNA transcript levels by real-time quantitative PCR

HepG2 cells (WT and PI31 KO) were seeded at 3×10^5^ cells/well on six-well plates and exposed to 100 U/ml IFNγ or medium controls for 24 h beginning the following day. After treatment, culture medium was aspirated, and cells were harvested in 1 ml ice-cold Trizol. Total RNA was isolated in the aqueous phase following two successive incubations with 200 µl chloroform. RNA was then precipitated with 500 µl isopropanol and washed three times with 1 ml 75% ethanol. Ethanol was then removed, and the pellet was air-dried before solubilization in nuclease-free water. RNA integrity was confirmed by SYBRSafe (Thermo Fisher Scientific, S33102) visualization of rRNA on 1% agarose gel, and RNA purity was assessed by a Nanodrop spectrophotometer (Thermo Scientific Nanodrop 2000C). Total RNA was stored at −80°C until use and thawed fewer than three times. First-strand cDNA was reverse transcribed using SuperScript III according to the manufacturer's instructions (Thermo Fisher Scientific, 18080044). 883 ng total RNA from each sample was used as a template with 50 ng random primers (Thermo Fisher Scientific, 48190011) in a total volume of 20 µl. First-strand cDNA was then diluted 20-fold with nuclease-free water.

Primer sequences for human transcripts for β1i (5′-CAACGTGAAGGAGGTCAGGTA-3′, 5′-AGAGCAATAGCGTCTGTGGTG-3′), β2i (5′-AATGTGGACGCATGTGTGAT-3′, 5′-CATAGCCTGCACAGTTTCCTC-3′), β5i (5′-CACGGGTAGTGGGAACACTTA-3′, 5′-ACTTTCACCCAACCATCTTCC-3′), PA28β (5′-CTTTTCCAGGAGGCTGAGG-3′, 5′-CGGAGGGAAGTCAAGTCA-3′) and β-actin (5′-CTGGACTTCGAGCAAGAGATG-3′, 5′- TGAAGGTAGTTTCGTGGATGC-3′) were derived from previous studies (Camargo et al., 2014; Morawietz et al., 2009) and synthesized for use in the present study by Sigma-Aldrich. Quantitative PCR reactions were carried out in 10 µl reactions using a 384-well format for the CFX Opus 84 Real-Time PCR System (Bio-Rad). 8.8 ng diluted cDNA templates were mixed with 0.2 µM primers and 2× Universal SYBR Green Fast qPCR Mix (ABclonal, RK21203) in triplicate. Targets were initially heated at 95°C for 3 min and then amplified with 40 cycles of 95°C (30 s) and 62°C (60 s). Following amplification, a melt curve was measured from 65°C to 95°C at 0.5°C intervals to confirm amplification product quality. Data were imported into CFX Maestro Software (Bio-Rad, v2.3) to determine Ct values. Relative quantifications (ΔCt) of genes were calculated relative to actin, and ΔΔCt was calculated with WT controls as the reference sample.

### Statistics

Experiments were repeated independently for the number of biological replicates indicated in figure legends. Treatment effects were analyzed by repeated measures ANOVA matched within experiments. Where significant treatment effects were found, Tukey's HSD post-hoc test was performed to determine pairwise differences. All statistics were calculated using GraphPad Prism Software (v10.2.3).

## Supplementary Material



10.1242/joces.263887_sup1 Supplementary information
