# Supplementary material for: PI31 is a positive regulator of 20S immunoproteasome assembly
Source: J Cell Sci. 2025 May 23;138(10):jcs263887. doi: 10.1242/jcs.263887 (PMC12148024; doi:10.1242/jcs.263887)
Supplement: Supplementary information [file joces-138-263887-s1.pdf]

A.

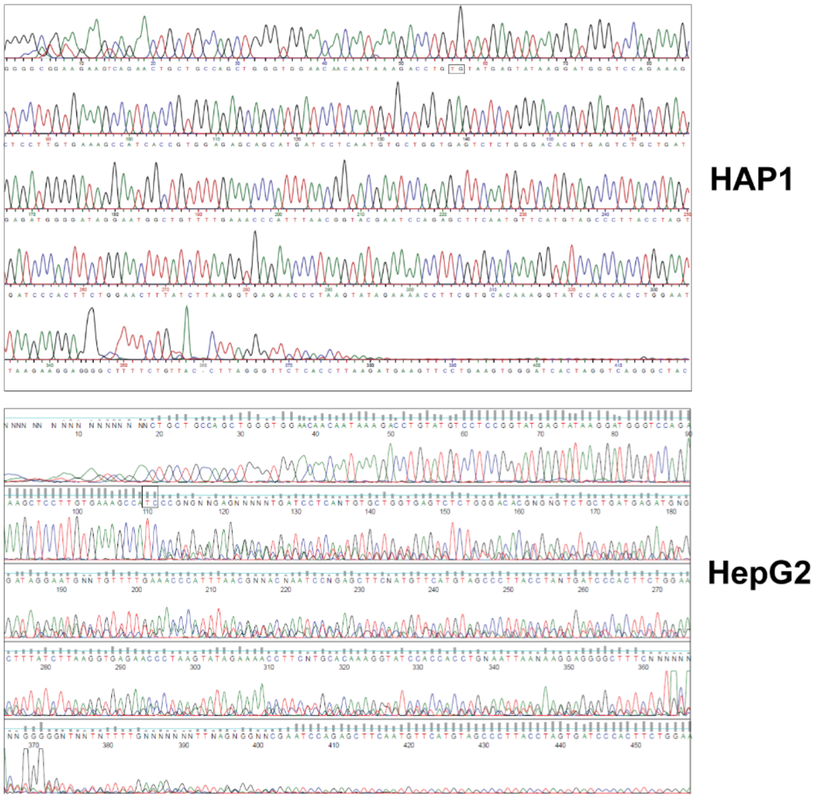

B.

```
                                    << Exon 2 >>                             |----gRNA (HAP1)----|
NC_000020.11:1125529-1125804        TGCCAGCTGGGTGGAACAACAATAAAGACCTGTATGTCCTCCGGTATGAGTATAAGGATG    60
HepG2-WT                            TGCCAGCTGGGTGGAACAACAATAAAGACCTGTATGTCCTCCGGTATGAGTATAAGGATG    60
HepG2-KO                            TGCCAGCTGGGTGGAACAACAATAAAGACCTGTATGTCCTCCGGTATGAGTATAAGGATG    60
HAP1-KO                             TGCCAGCTGGGTGGAACAACAATAAAGACCTGT----------GTATGAGTATAAGGATG    50
                                    *********************************          *****************

                                                 |---gRNA (HepG2)----|
NC_000020.11:1125529-1125804        GGTCCAGAAAGCTCCTTGTGAAAGCCATCACCGTGGAGAGCAGCATGATCCTCAATGTGC    120
HepG2-WT                            GGTCCAGAAAGCTCCTTGTGAAAGCCATCACCGTGGAGAGCAGCATGATCCTCAATGTGC    120
HepG2-KO                            GGTCCAGAAAGCTCCTTGTGAAAGCCATC-CCGTGGAGAGCAGCATGATCCTCAATGTGC    119
HAP1-KO                             GGTCCAGAAAGCTCCTTGTGAAAGCCATCACCGTGGAGAGCAGCATGATCCTCAATGTGC    110
                                    ***************************** ******************************

                                     |Intron >>
NC_000020.11:1125529-1125804        TGGTGAGTCTCTGGGACACGTGAGTCTGCTGATGAGATGGGGATAGGAATGGCTGTTTTG    180
HepG2-WT                            TGGTGAGTCTCTGGGACACGTGAGTCTGCTGATGAGATGGGGATAGGAATGGCTGTTTTG    180
HepG2-KO                            TGGTGAGTCTCTGGGACACGTGAGTCTGCTGATGAGATGGGGATAGGAATGGCTGTTTTG    179
HAP1-KO                             TGGTGAGTCTCTGGGACACGTGAGTCTGCTGATGAGATGGGGATAGGAATGGCTGTTTTG    170
                                    ************************************************************

NC_000020.11:1125529-1125804        AAACCCATTTAACGGTACGAATCCAGAGCTTCAATGTTCATGTAGCCCTTACCTAGTGAT    240
HepG2-WT                            AAACCCATTTAACGGTACGAATCCAGAGCTTCAATGTTCATGTAGCCCTTACCTAGTGAT    240
HepG2-KO                            AAACCCATTTAACGGTACGAATCCAGAGCTTCAATGTTCATGTAGCCCTTACCTAGTGAT    239
HAP1-KO                             AAACCCATTTAACGGTACGAATCCAGAGCTTCAATGTTCATGTAGCCCTTACCTAGTGAT    230
                                    ************************************************************

NC_000020.11:1125529-1125804        CCCACTTCTGGAACTTTATCTTAAGGTGAGAACCCT    276
HepG2-WT                            CCCACTTCTGGAACTTTATCTTAAGGTGAGAACCCT    276
HepG2-KO                            CCCACTTCTGGAACTTTATCTTAAGGTGAGAACCCT    275
HAP1-KO                             CCCACTTCTGGAACTTTCTCTTAAGGTGAGAACCCT    266
                                    ***************** ******************
```

Fig. S1. Validation of PI31 knockout from HAP1 and HepG2 cells. The PSMF1 gene encoding PI31 was disrupted as described in Materials and Methods. **(Panel A),** Sanger sequencing results for PI31 knockout lines. (Upper), PI31 KO in HAP1 cells resulted from a 10 bp deletion between the indicated T and G nucleotides. (Lower), PI31-KO in HepG2 cells resulted from a 1 bp deletion after the indicated T and C nucleotides. Deletions are indicated by boxes. **(Panel B),** DNA sequences were aligned with the reference genome and the independently sequenced HepG2 wildtype sequence using Clustal Omega tool (https://www.ebi.ac.uk/jdispatcher/msa/clustalo). The boundary between the PSMF1 exon 2 and intron region is shown. Guide RNAs used to knockout PI31 from respective cell lines are underlined, and deleted sequences are highlighted in yellow.

Journal of Cell Science • Supplementary information

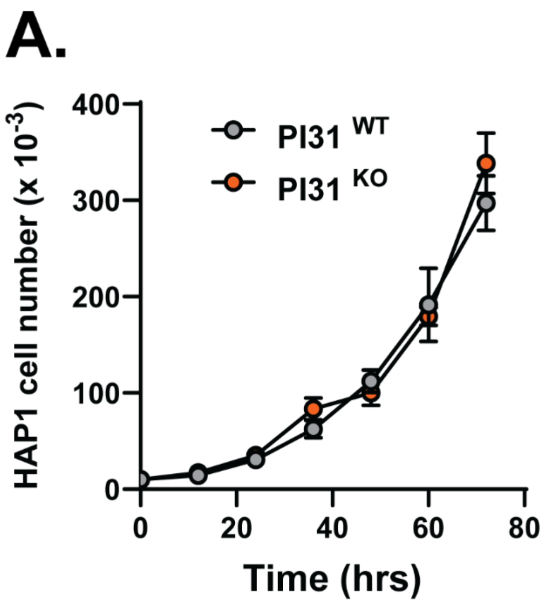

Fig. S2. PI31 knockout does not affect cell proliferation. PI31 wild-type and knockout HAP1 **(Panel A)** and HepG2 **(Panel B)** cells were seeded at 10,000 cells per plate and grown under standard culture conditions. Culture media was changed every 24 hrs. At indicated times, cells were harvested and counted. Data points represent mean cell number ± s.d. of triplicate plates.

Journal of Cell Science • Supplementary information

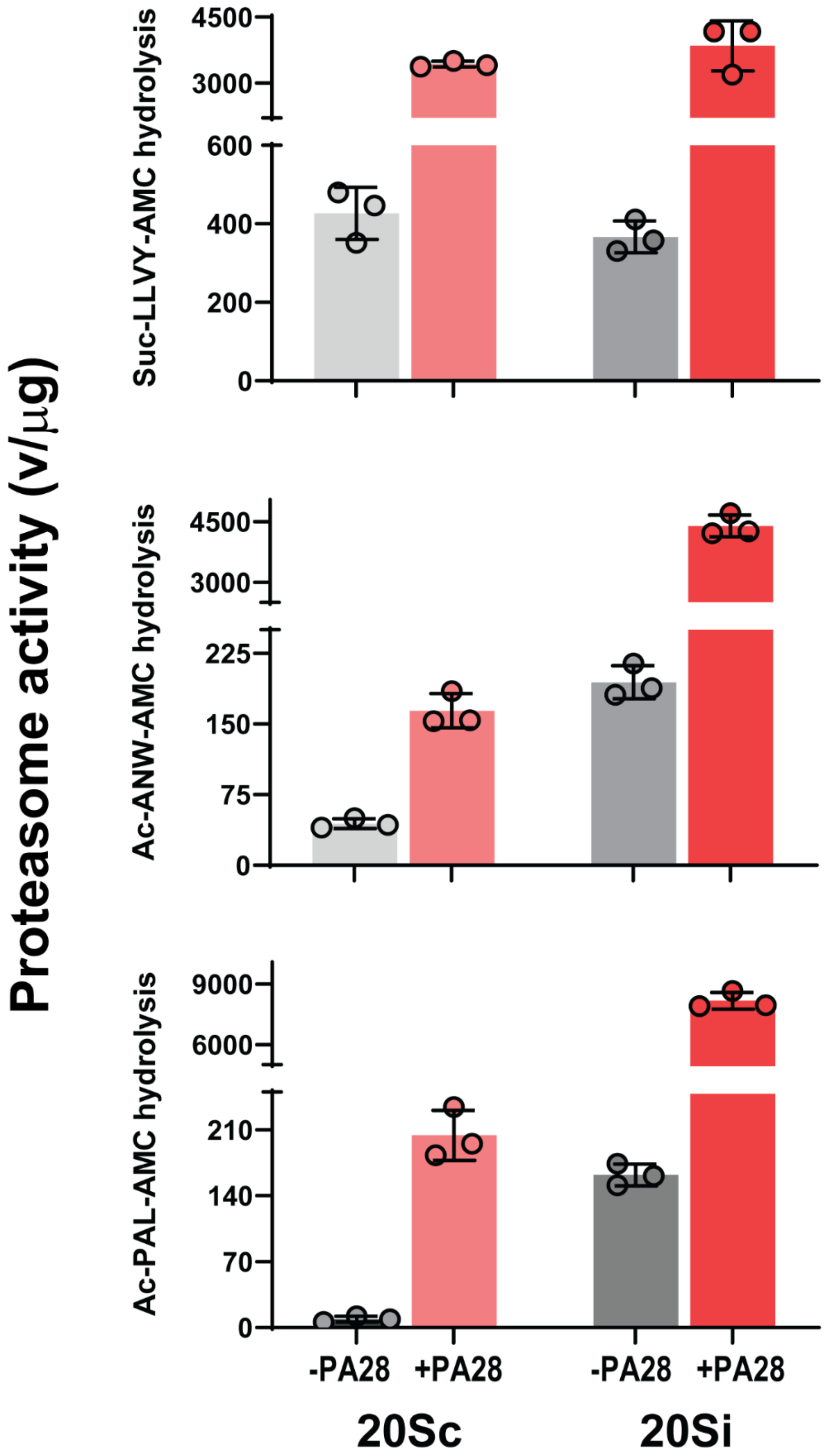

Fig. S3. Relative substrate specificities of constitutive and immuno- 20S proteasomes. Constitutive (20Sc) and immuno- 20S (20Si) proteasomes were purified from bovine red blood cells or bovine spleen, respectively, and assayed for hydrolysis of indicated peptide substrates in the presence or absence of purified PA28,βα as described previously (Wang *et al*, 2024). Bars represent mean rates of hydrolysis ± s.d. of triplicate assays. Similar results were obtained with two independent preparations of purified proteasomes.

Journal of Cell Science • Supplementary information

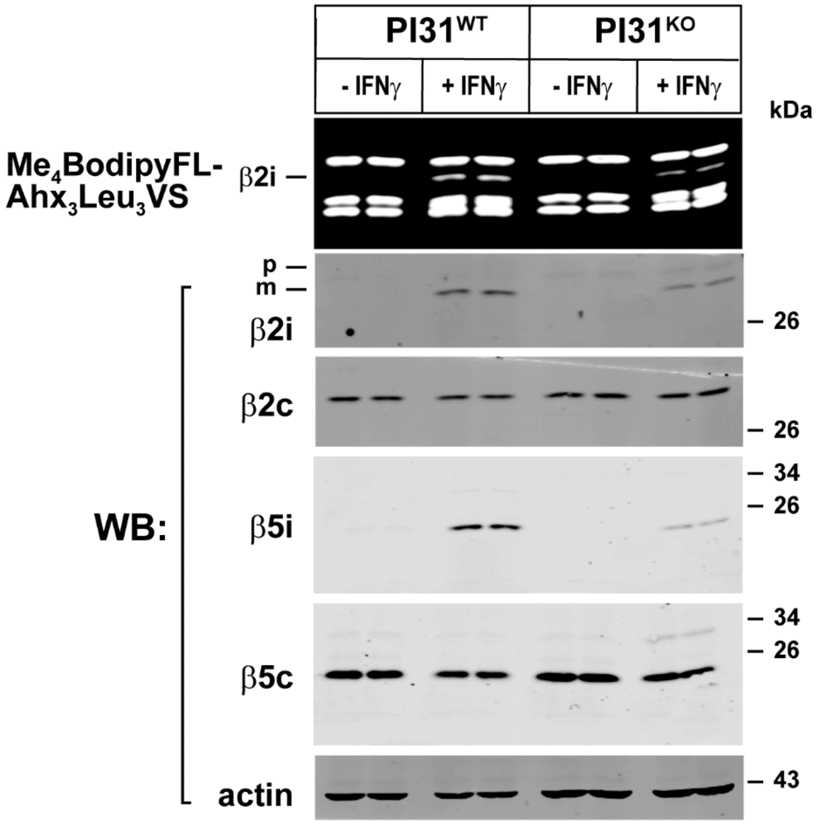

Fig. S4. PI31 knockout cells have attenuated activation of β2i activity upon treatment with interferon-.γ Wild-type and PI31 KO HAP1 cells were cultured in the absence (-IFNγ) or presence (+IFNγ) of 100 U/ml human interferon-γ for 24 hrs. Cell extracts were treated with Me_4BodipyFL-Ahx_3Leu_3VS and analyzed as described under Materials and Methods. Equal amounts of extract protein from two independent experiments were subjected to SDS-PAGE and imaged for activity probe or subjected to Western blotting for indicated proteins. The position of propeptide and mature forms of β2i are indicated by “p” and “m”, respectively.

Journal of Cell Science • Supplementary information

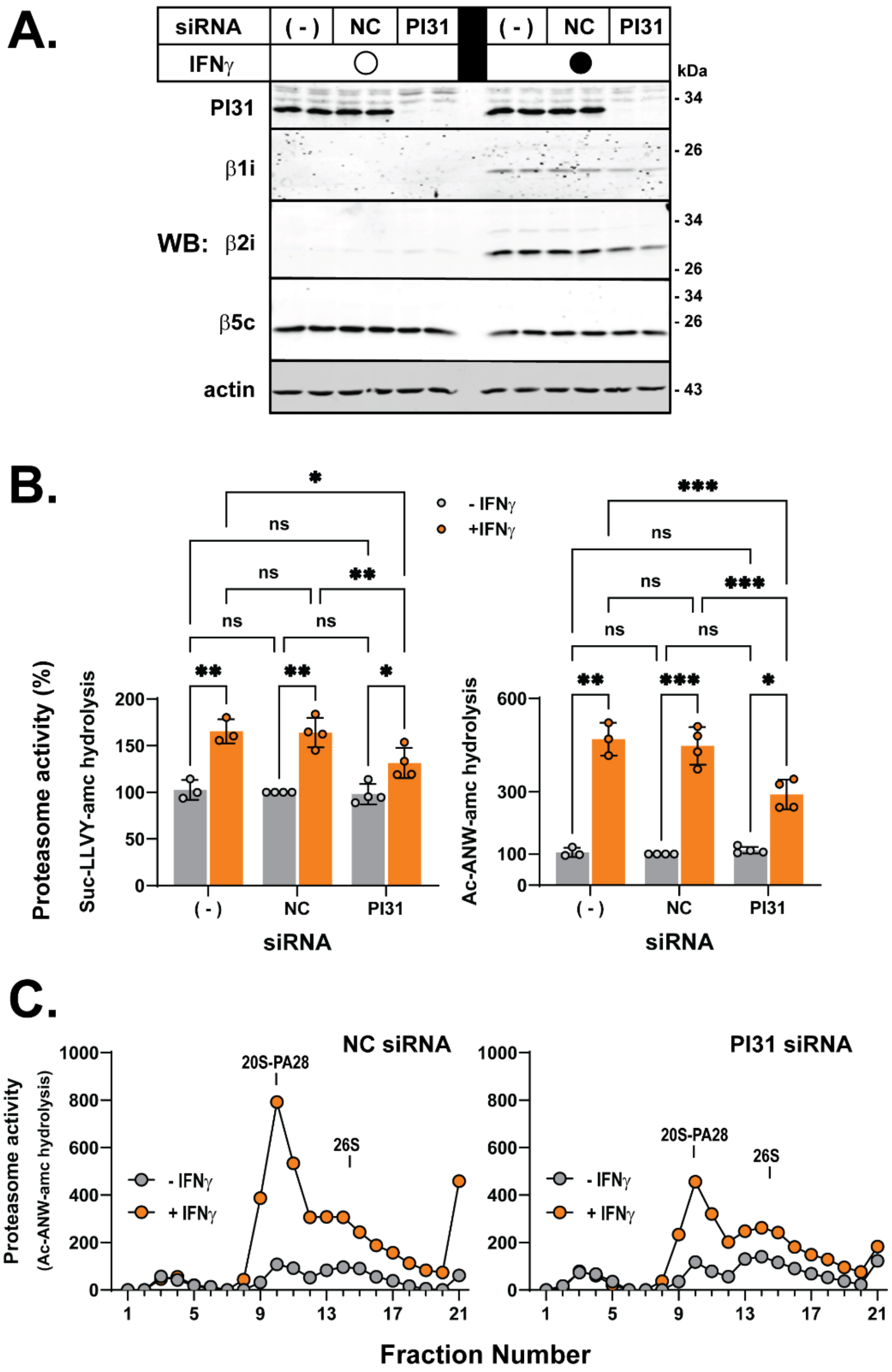

Journal of Cell Science • Supplementary information

Fig. S5. Knockdown of PI31 by RNAi inhibits interferon-γ induced increases of immunoproteasome content and activity in HepG2 cells. HepG2 cells were transfected with a non-coding siRNA (NC), an siRNA targeting PI31 (PI31), or transfection reagent only (-). After 24 hrs, cells were treated with 100 U/ml of interferon-γ for 48 hrs. Cells extracts were prepared as described under Materials and Methods. (**Panel A),** Cell extracts for indicated conditions were normalized for total protein and subjected to western blotting for indicated proteins. Lanes show blots from two independent biologic experiments. **(Panel B**), Extracts, normalized for total protein were assayed for proteasome activity using the indicated peptide substrates. Rates of substrate hydrolysis for cells treated with non-coding (NC) siRNA were set to 100 and rates for all other treatments were expressed relative to that value. Each data point represents the mean value of triplicate assays for a given biologic experiment. Bars represent mean values ± s.d. of independent biologic experiments. Differences were analyzed by repeated measures 2-way ANOVA and Tukey's HSD posthoc test (* $p<0.05$; ** $p<0.01$; *** $p<0.001$). **(Panel C)**, Extracts of cells from indicated conditions were normalized for total protein and subjected to glycerol density gradient centrifugation as described in Materials and Methods. Gradient fractions were assayed for immuno-proteasome activity using Ac-ANW-amc substrate. 20S-PA28 and 26S show sedimentation positions of respective purified holoenzyme standards.

Journal of Cell Science • Supplementary information

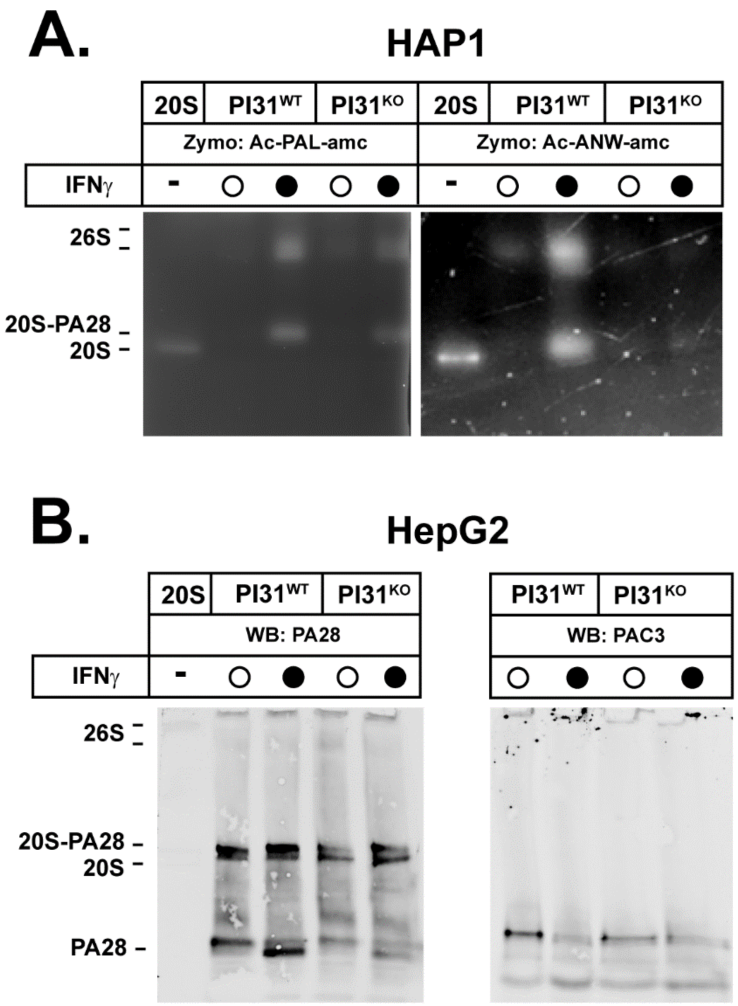

Fig. S6. PI31 knockout cells have attenuated activities of immunoproteasome holoenzymes upon treatment with interferon-.γ PI31 WT and PI31 KO HAP1 and HepG2 cells were exposed to 100 U/ml human IFNγ (●) or control buffer (O) for 24 hours. Cell extracts were normalized for total protein and subjected to native PAGE for zymography with indicated proteasome substrates **(Panel A)** or Western blotting for indicated proteins **(Panel B)**. Purified 20S proteasome (20S) was electrophoresed as a standard, as indicated.

Journal of Cell Science • Supplementary information

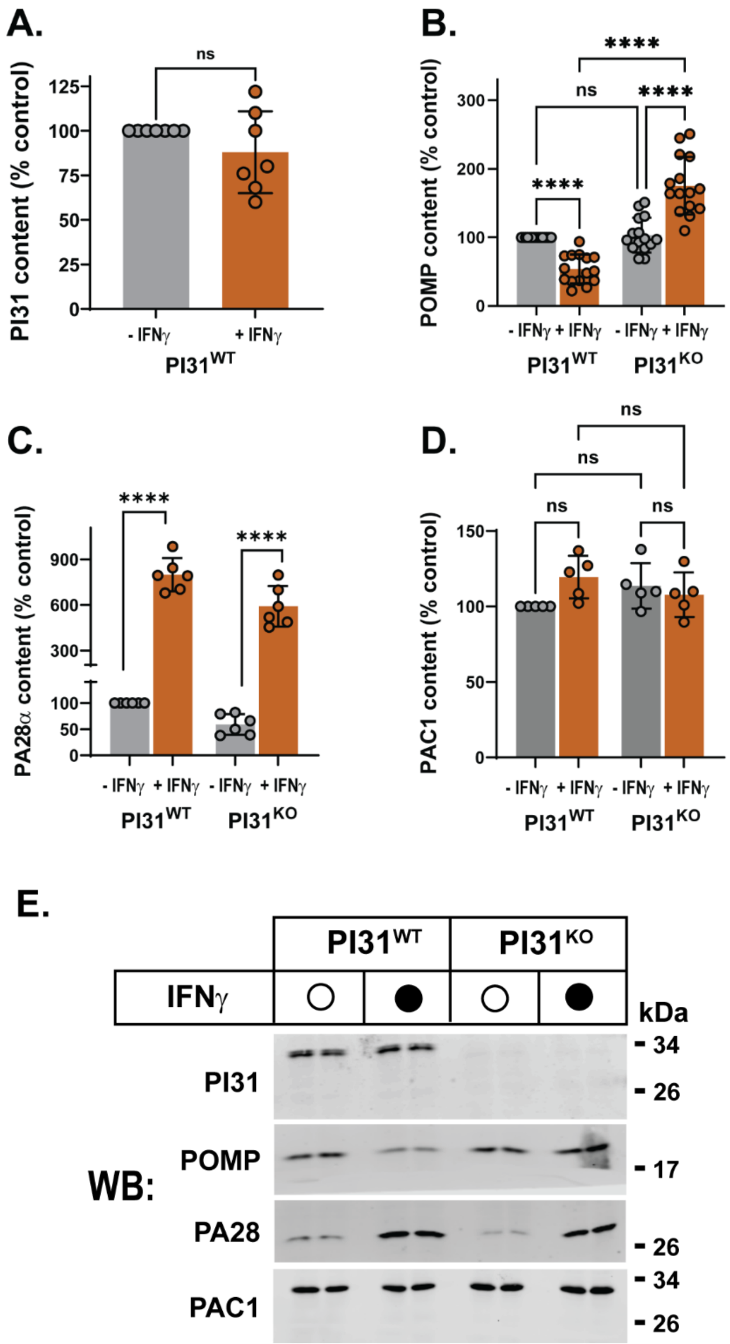

Fig. S7. Effect of PI31 knockout on regulatory proteins of the proteasome. PI31 wild-type and PI31 KO HAP1 cells were cultured in the absence (-IFNγ) or presence (+IFNγ) of 100 U/ml human interferon-γ for 24 hrs as indicated. Extracts were prepared as described in Materials and Methods, normalized for total protein and subjected to Western blotting for indicated proteins. Blots for PI31 **(Panel A)**, POMP **(Panel B)**, PA28α **(Panel C)**, and PAC1 **(Panel D)** were quantified using ImageStudio (LiCOR) software. Within each independent experiment, the protein level from PI31 WT cells in the absence of interferon-γ was set at a value of 100 and all other values were expressed relative to that. Individual data points represent independent biologic experiments. Differences were analyzed by repeated measures 1- or 2-way ANOVA and Tukey's HSD posthoc test where appropriate (*ns* $p>0.05$; **** $p<0.0001$). **Panel E.** Extracts from representative experiments were subjected to Western blotting for indicated proteins. For given proteins, equal protein loads were analyzed.

Journal of Cell Science • Supplementary information

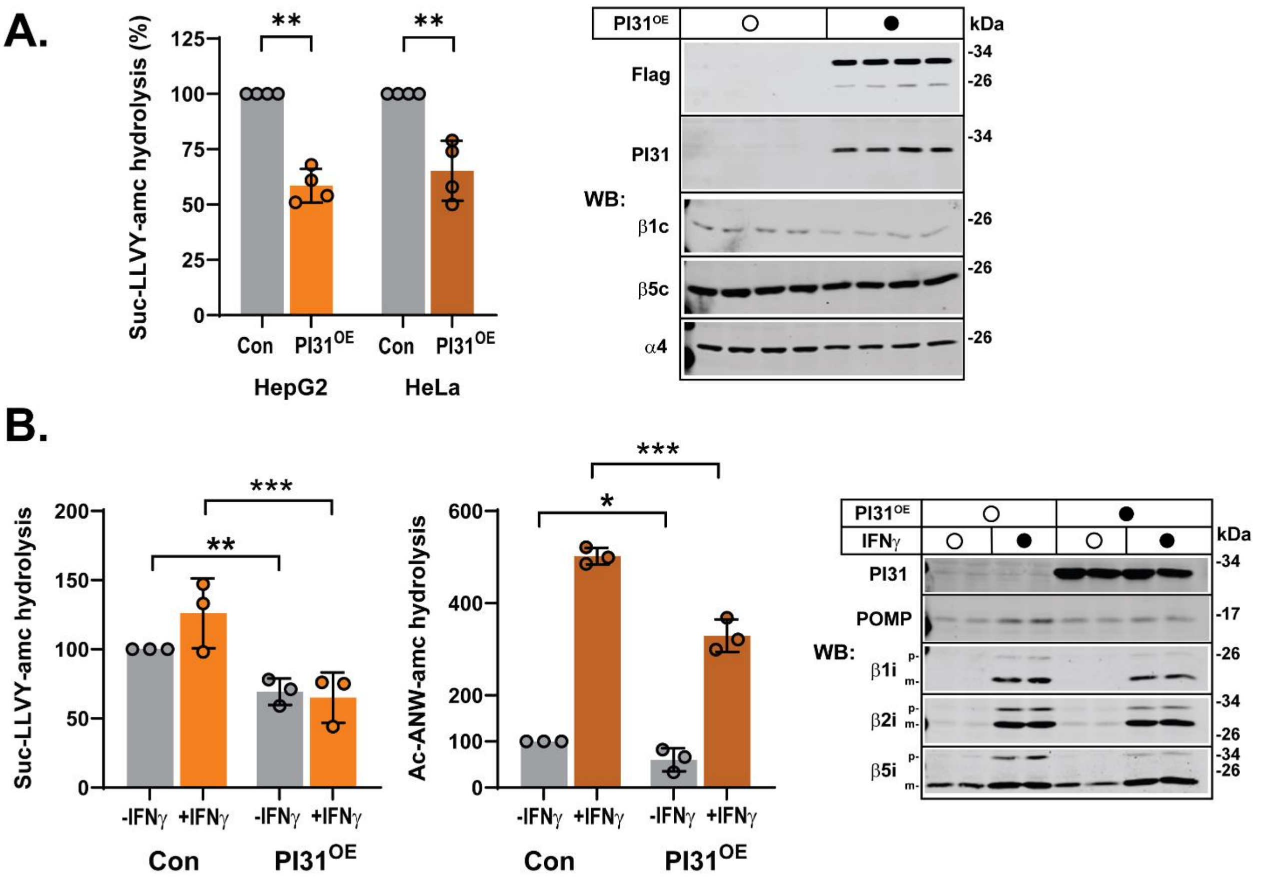

Fig. S8. Massive overexpression of PI31 inhibits constitutive and IFNγ-induced immuno-proteasome activities in HepG2 and HeLa cells. HepG2 and HeLa cells were transfected with Flag-PI31 (PI31^{OE}) or transfection reagent (Con) as described in Materials and Methods. **(Panel A),** 48 hrs after transfection extracts of HepG2 or HeLa cells were normalized for total protein and assayed for constitutive proteasome activity (left) or subjected to western blotting for indicated proteins (right). Bars represent mean values ± s.d. of indicated independent experiments. Control values were assigned values of 100 and PI31 overexpression samples were expressed relative to that. Differences were analyzed by repeated measures ANOVA and Tukey's HSD posthoc test ** $p<0.01$. Western blots show HeLa cell extracts from four independent experiments. **(Panel B),** HeLa cells were transfected with Flag-PI31 or vector alone. After 24 hrs, indicated cells were treated with 100 U/ml interferon-γ for an additional 24 hrs. Cell extracts were assayed for proteasome activity with the indicated substrates (left) or subjected to western blotting for the indicated proteins (right). Rates of substrate hydrolysis for cells treated with vector alone were set to 100 and rates for all other treatments were expressed relative to that value. Each data point represents the mean value of triplicate assays for a given biologic experiment. Bars represent mean values ± s.d. of independent biologic experiments. Differences were analyzed by repeated measures 2-way ANOVA and Tukey's HSD posthoc test (* $p<0.05$; ** $p<0.01$; *** $p<0.001$). Western blots show data from two independent biologic experiments. "p-" and "m" indicate positions of the unprocessed pro-peptide and mature forms, respectively, of indicated βi subunits.

Journal of Cell Science • Supplementary information

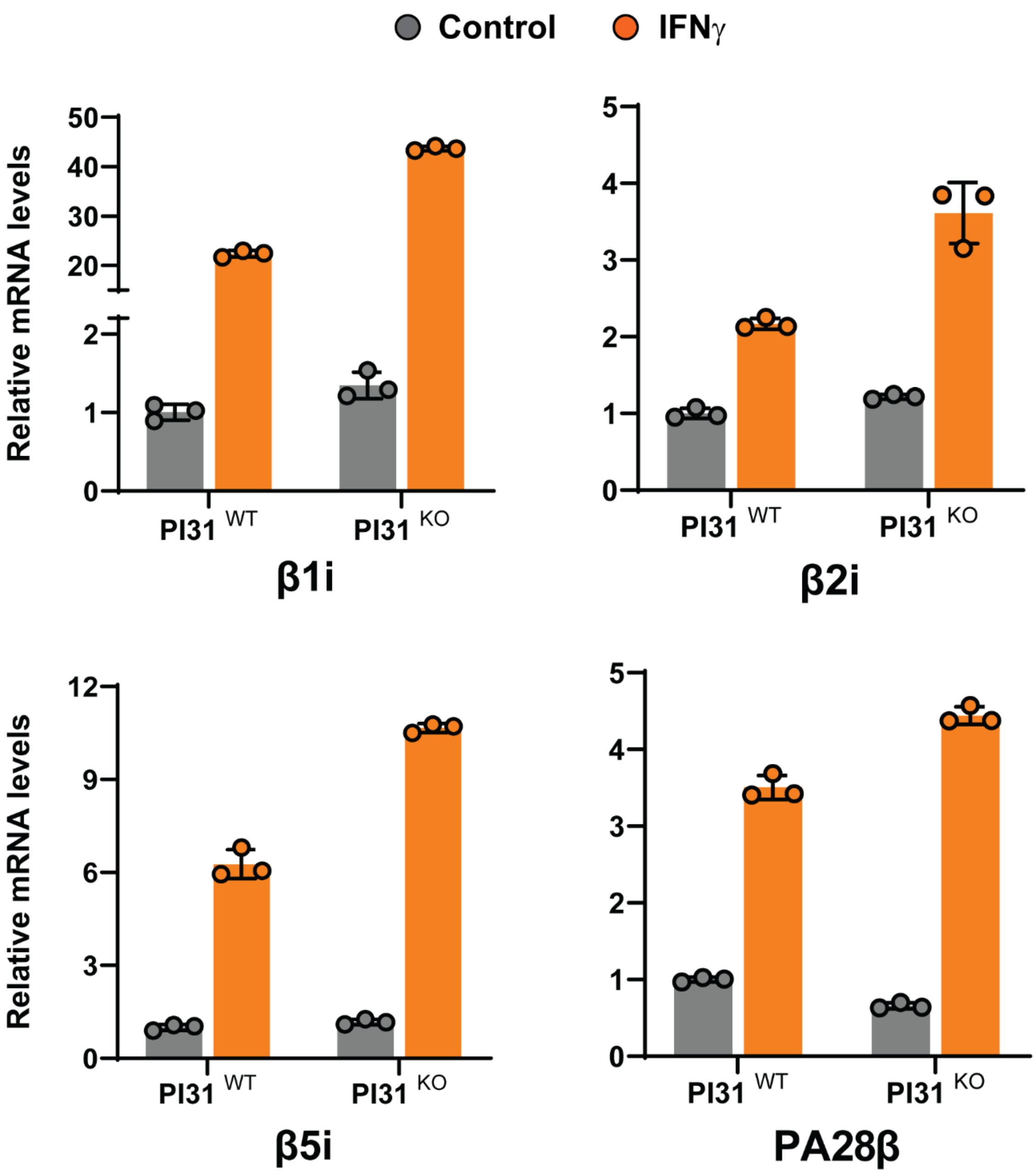

Fig. S9. PI31 wild-type and knockout cells have similar mRNA levels of βi subunits after interferon-γ treatment. PI31 wild-type and PI31 KO HepG2 cells were cultured in the absence (-IFNγ) or presence (+IFN γ) of 100 U/ml human interferon-γ for 24 hrs as indicated. Cell extracts were subjected to real time qPCR as described in Materials and Methods. Transcript expression levels are expressed as $2^{-\Delta\Delta Ct}$ normalized to β-actin as a reference gene and wild-type control as the reference sample. Bars show the mean ± s.d. of triplicate measurements.

Journal of Cell Science • Supplementary information

Blots for Figure 1.

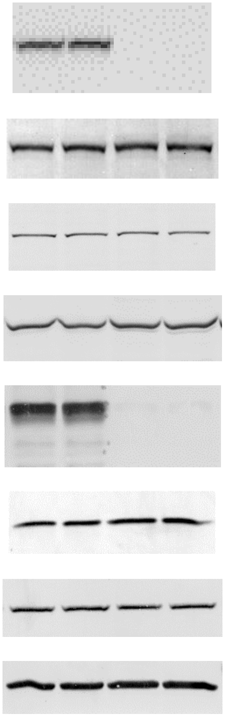

Journal of Cell Science • Supplementary information

Figure 1D

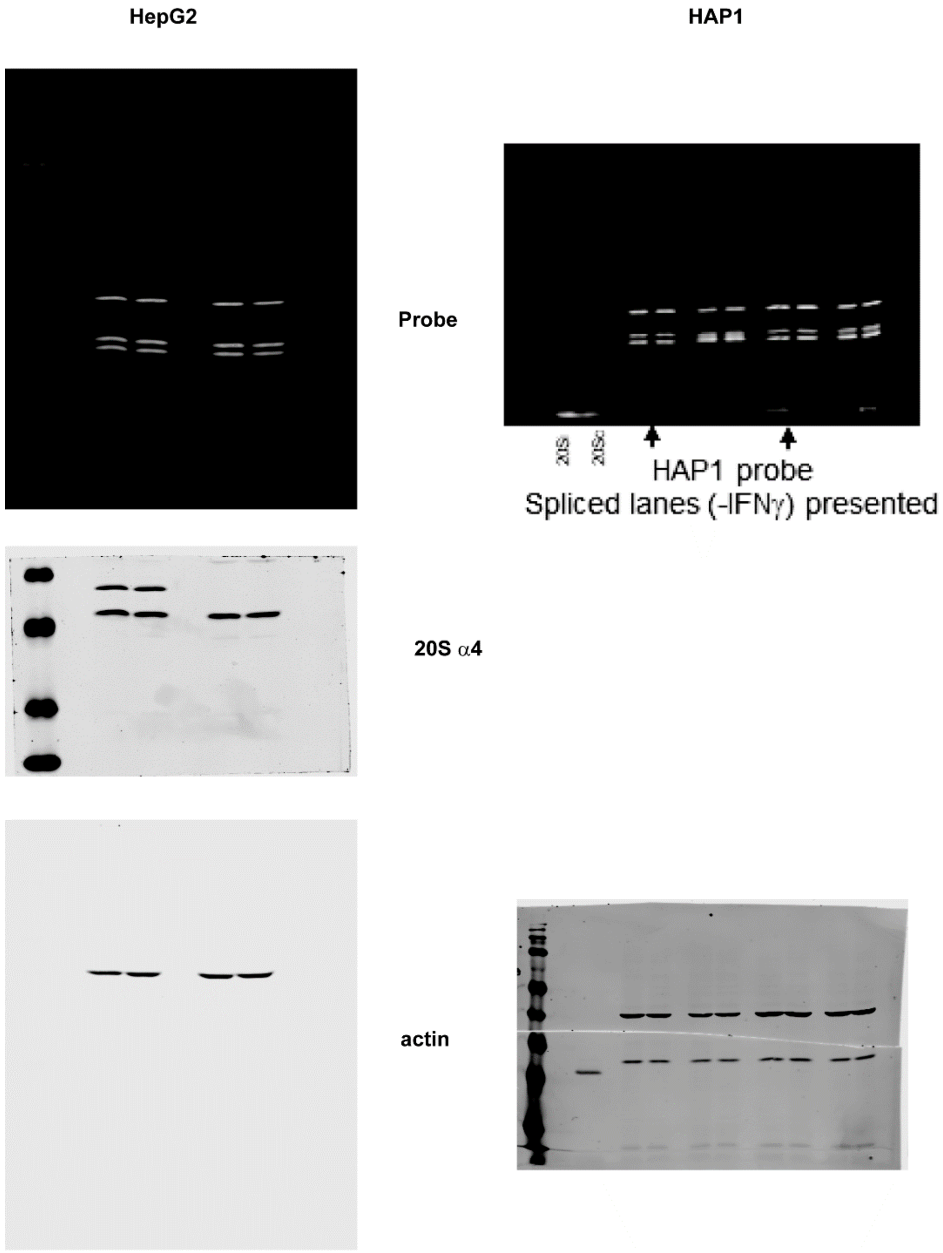

Journal of Cell Science • Supplementary information

Figure 1E

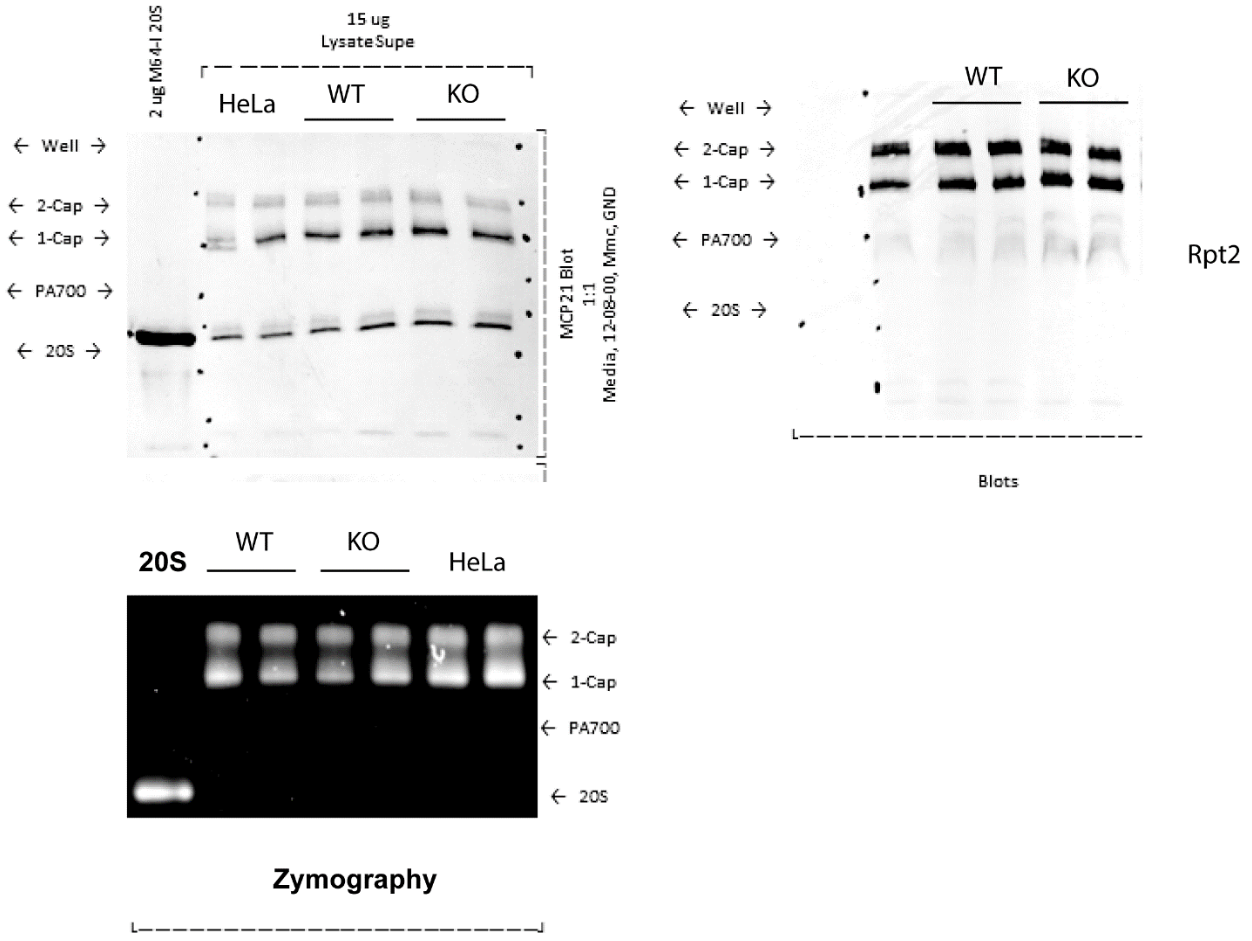

HAP1 and HeLa lysates

Journal of Cell Science • Supplementary information

Figure 2

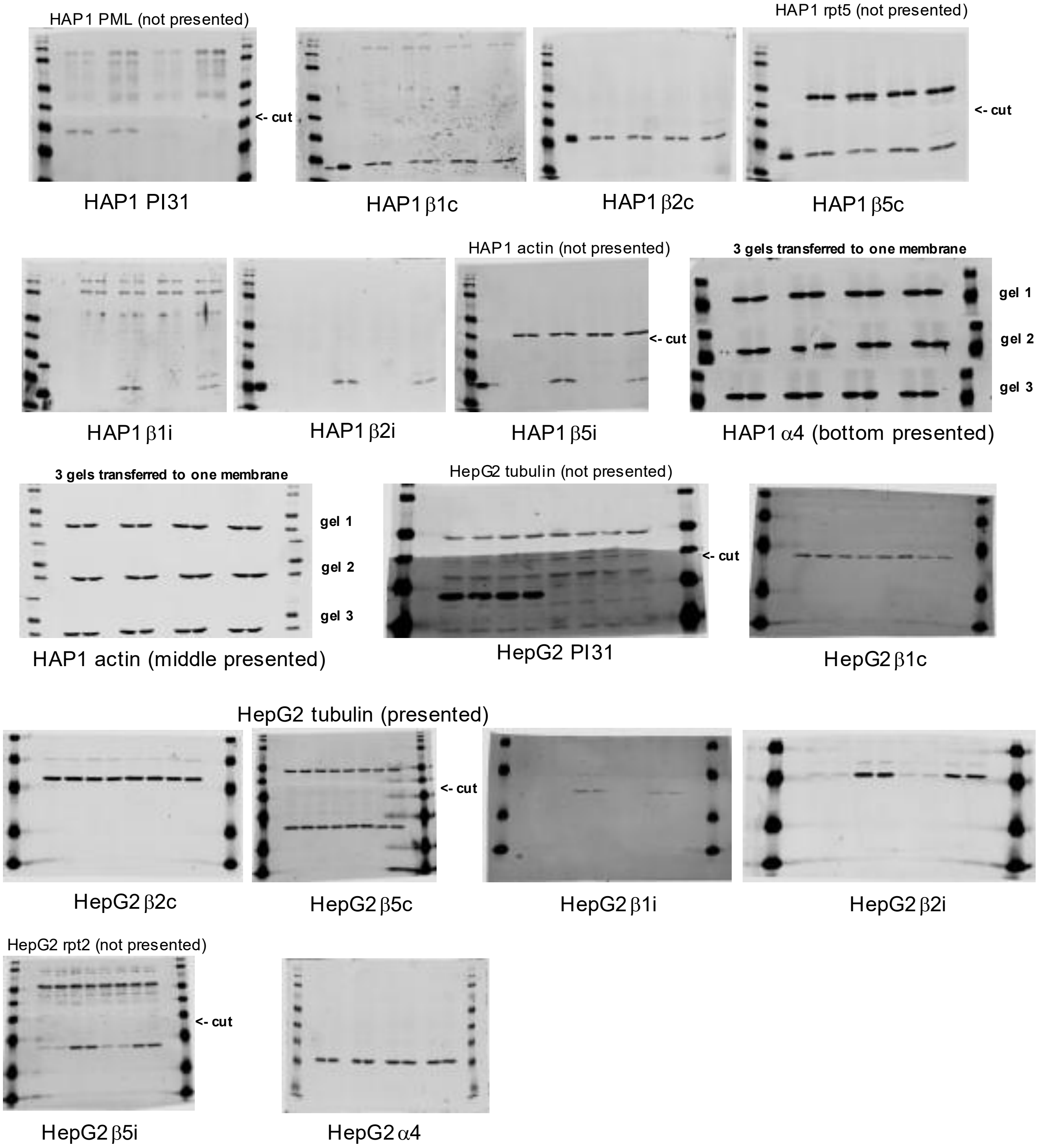

Journal of Cell Science • Supplementary information

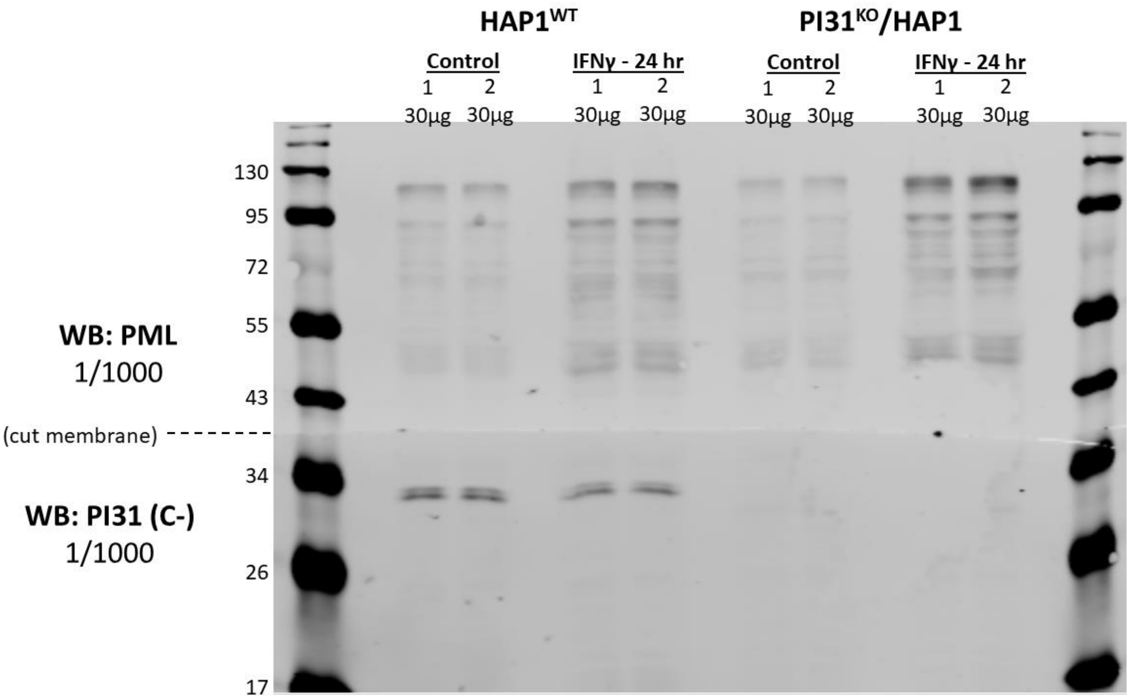

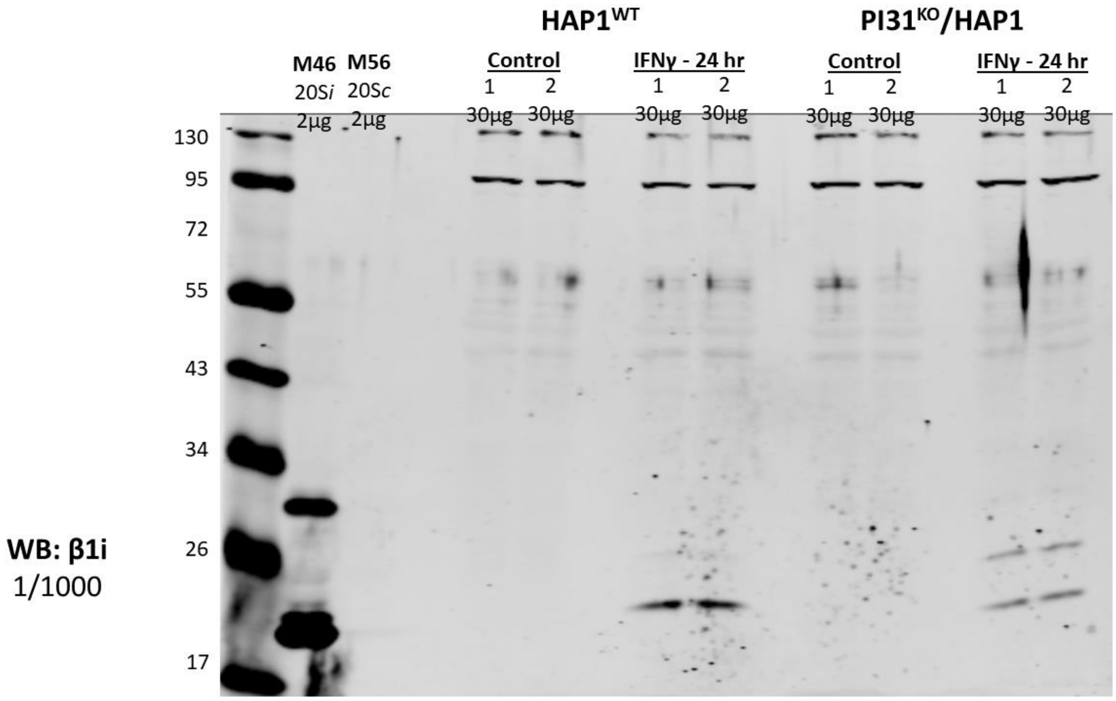

Journal of Cell Science • Supplementary information

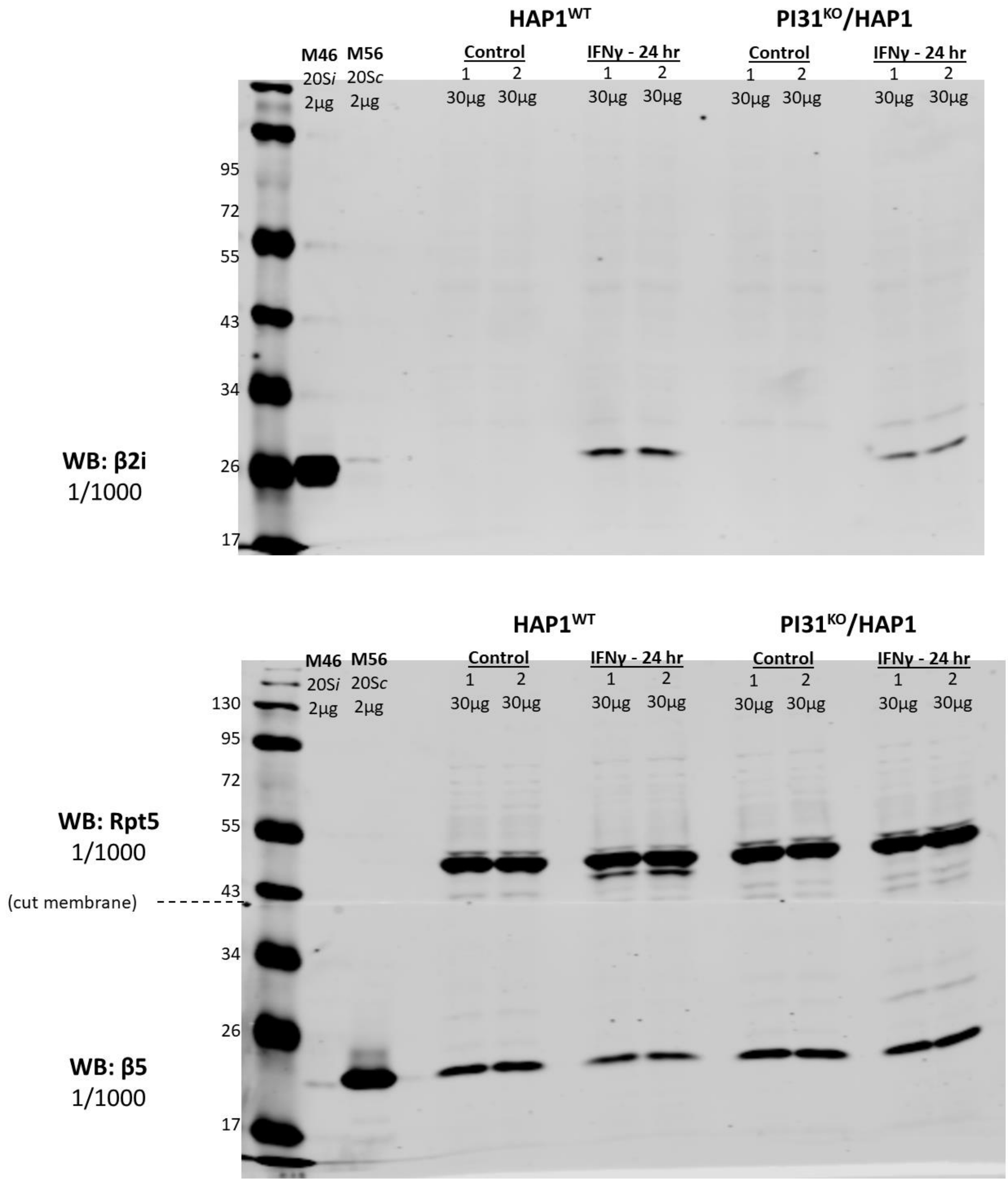

Journal of Cell Science • Supplementary information

Figure 4

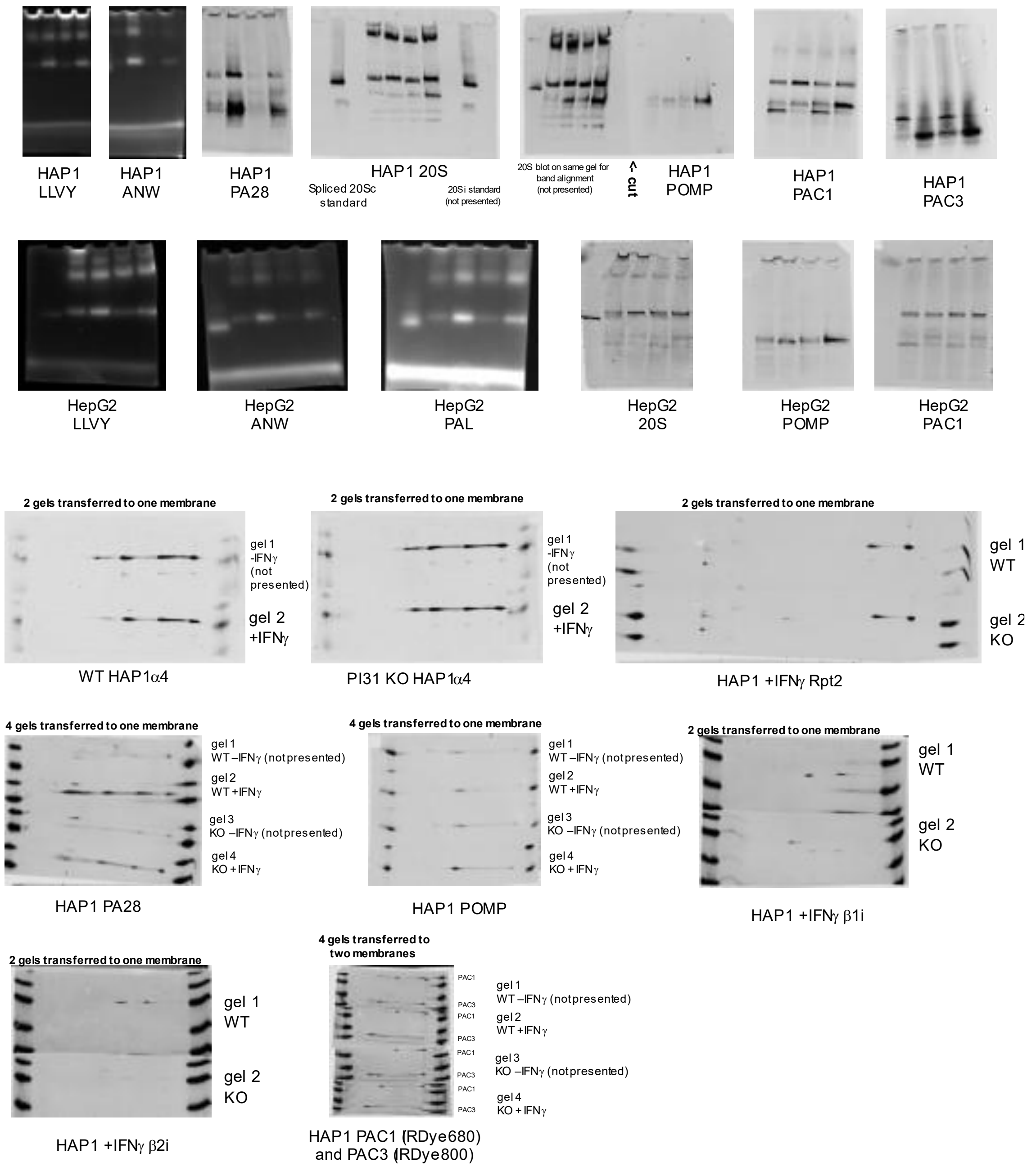

Journal of Cell Science • Supplementary information

Figure 5B

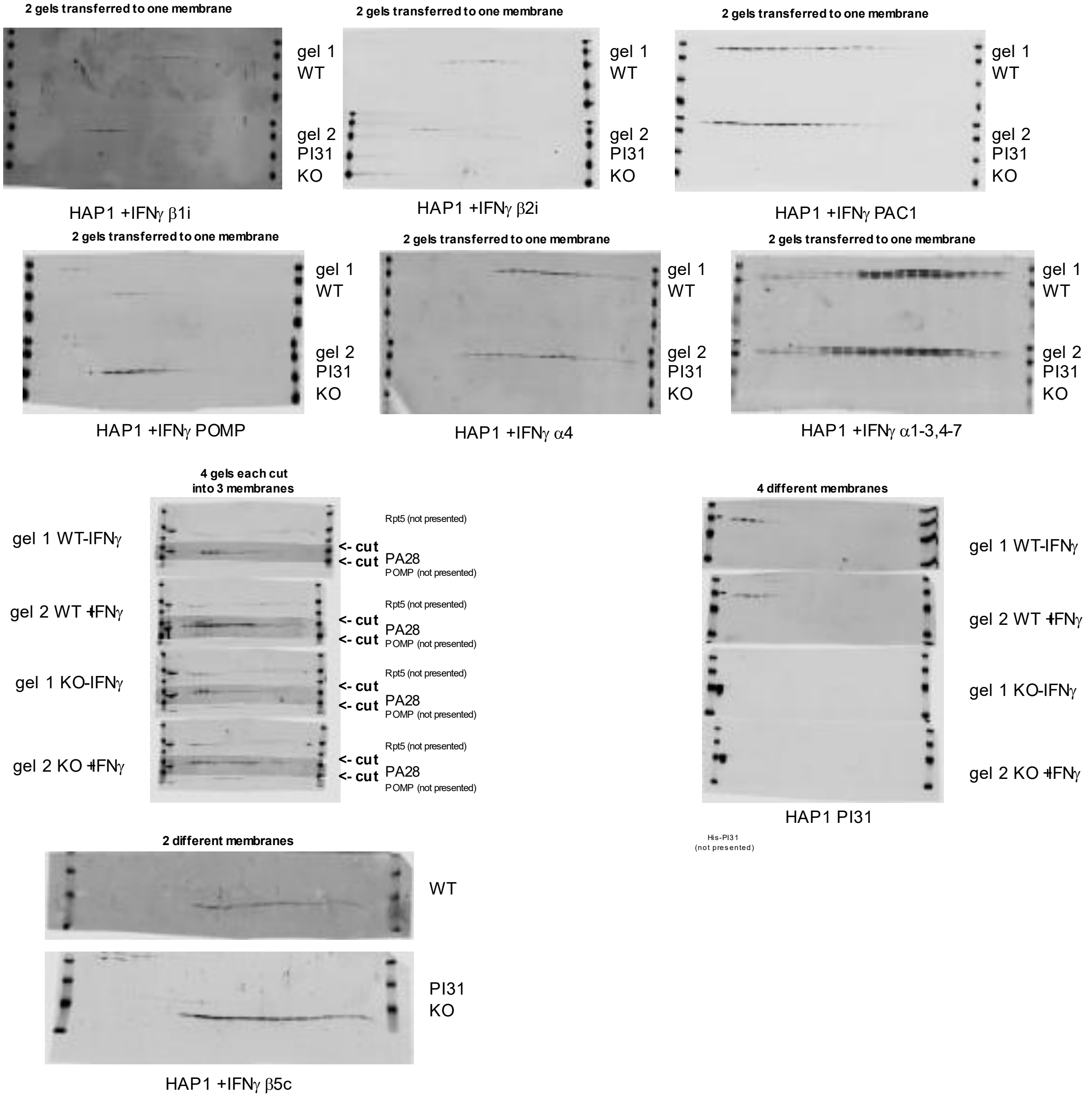

Journal of Cell Science • Supplementary information

Figure 6A

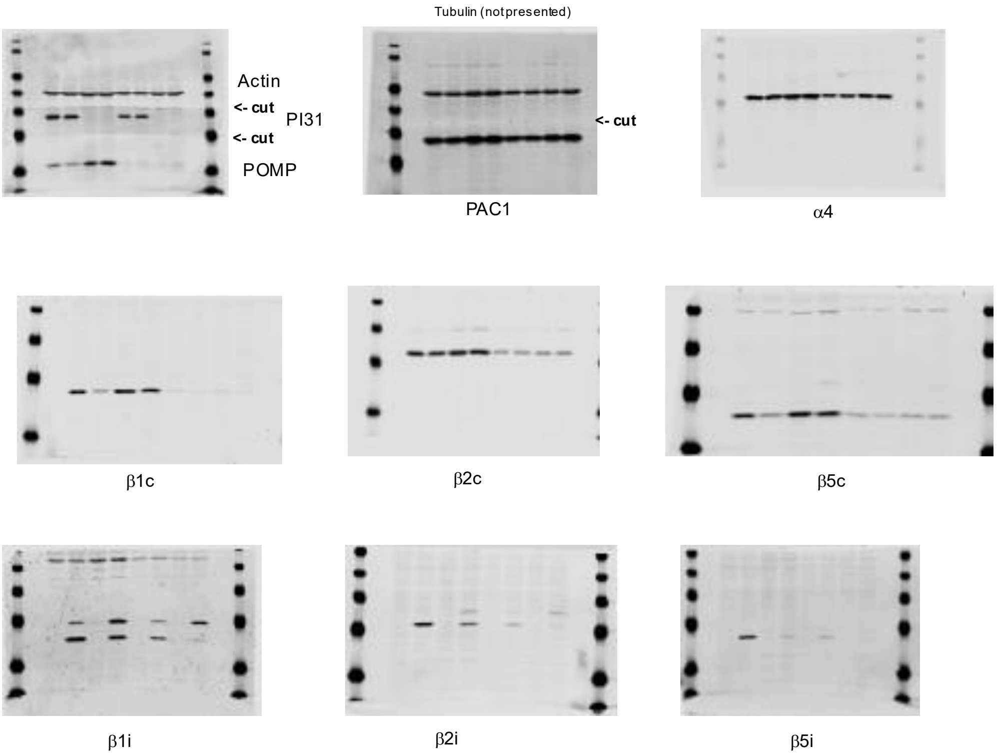

Figure 6C

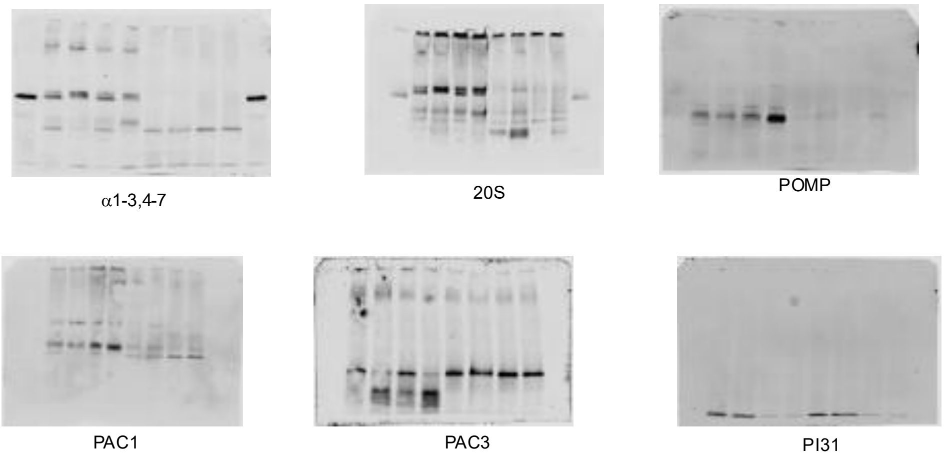

Journal of Cell Science • Supplementary information

Figure S4

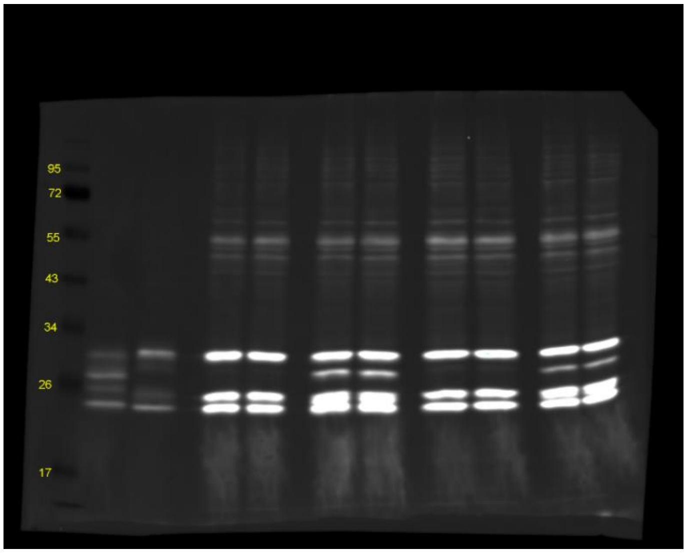

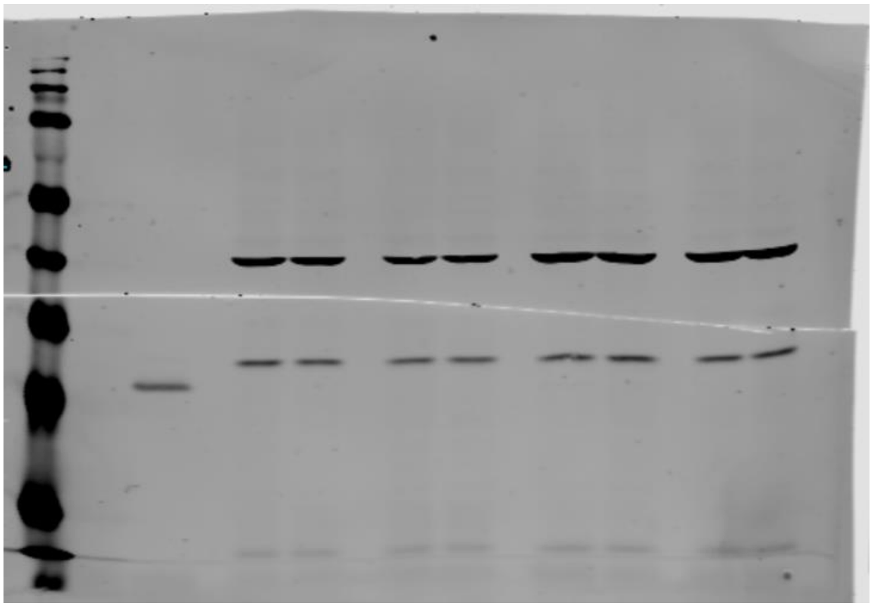

Journal of Cell Science • Supplementary information

Figure S4

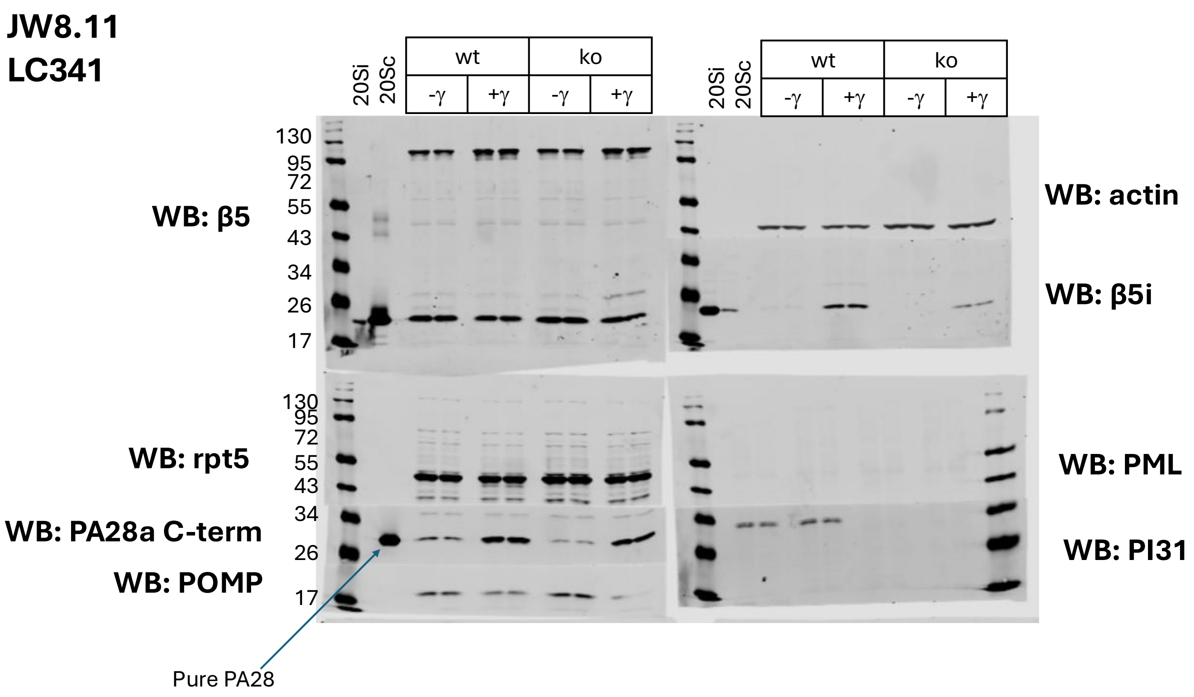

Journal of Cell Science • Supplementary information

Figure S5A

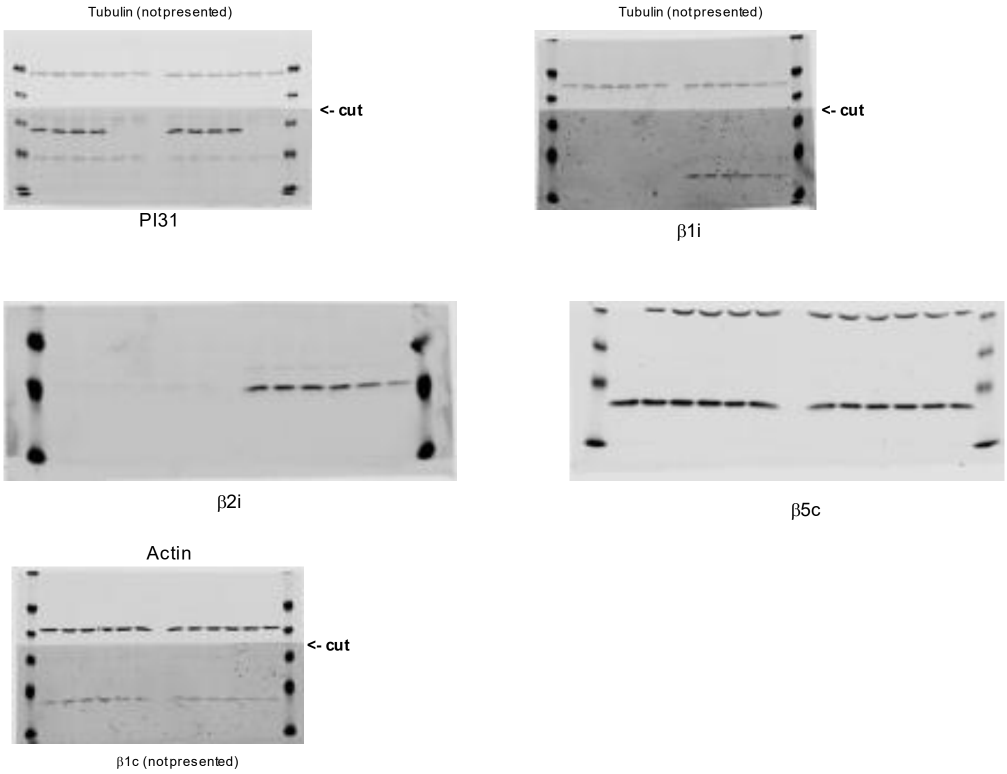

Journal of Cell Science • Supplementary information

Figure S6

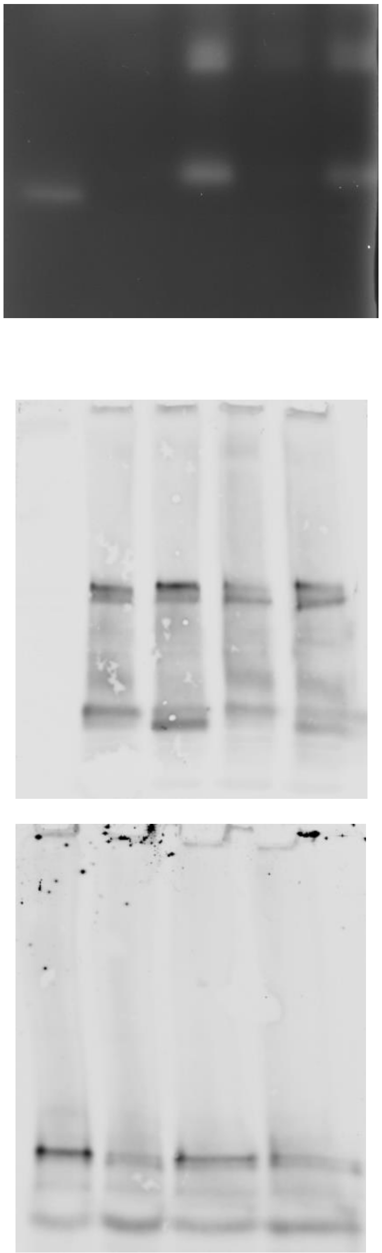

Journal of Cell Science • Supplementary information

Figure S8A

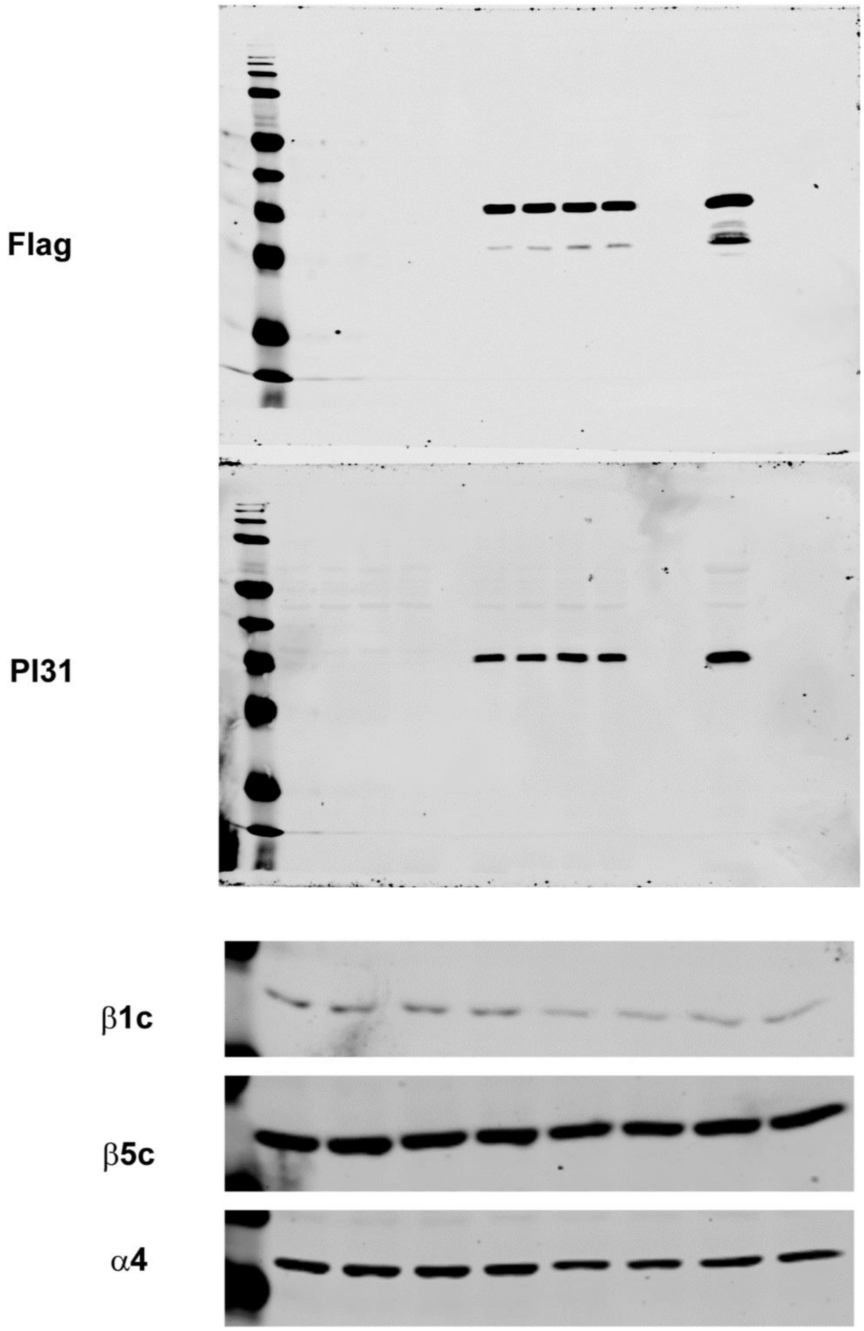

Journal of Cell Science • Supplementary information

Figure S8B

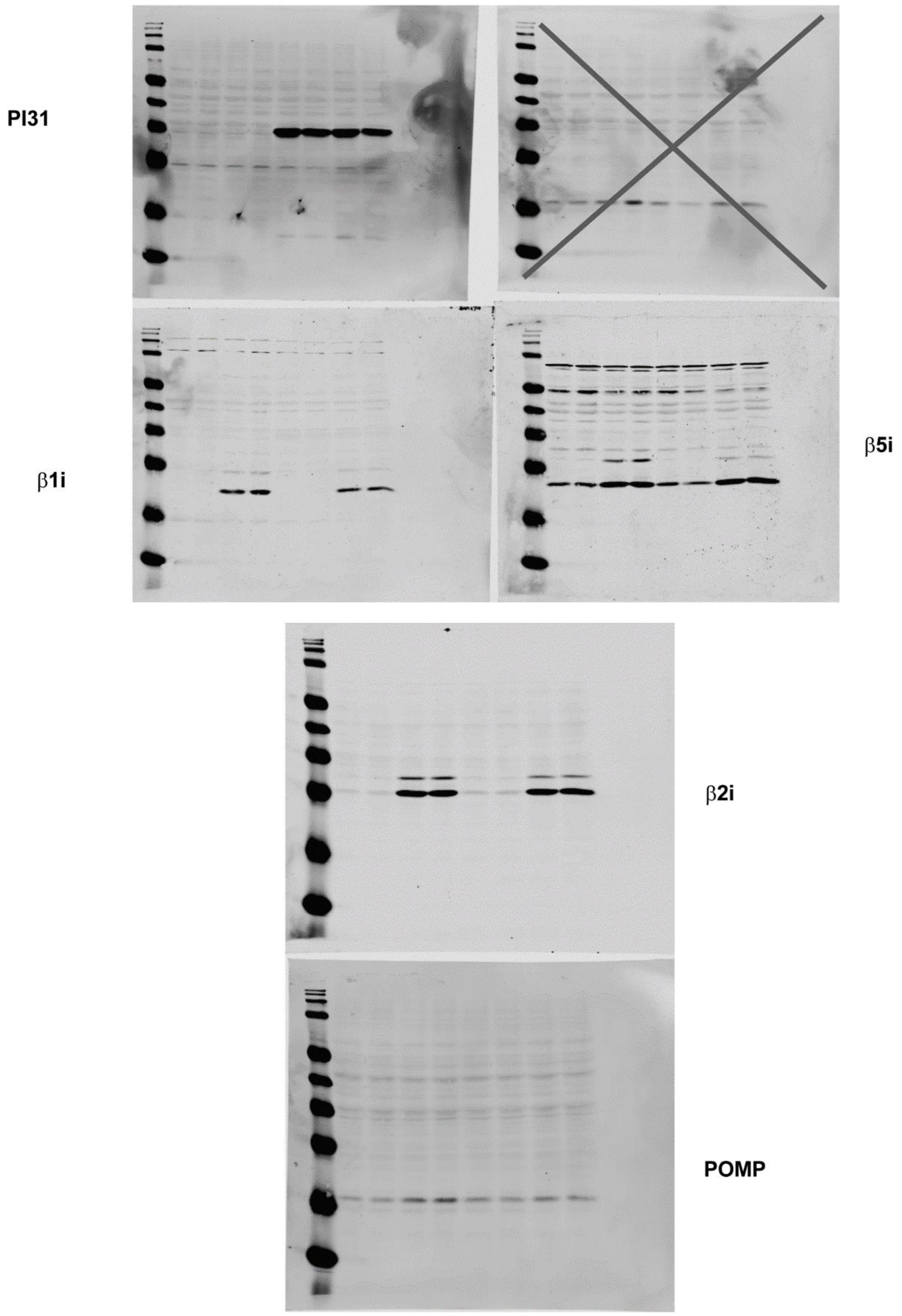

Fig. S10. Original Western blots. Original Western blots are presented as uncropped images. In some cases, blots for entire intact membranes are unavailable because membranes were cut to allow probing of mulitple proteins with corresponding antibodies.

Journal of Cell Science • Supplementary information
