# Supplementary material for: PI31 is a positive regulator of 20S immunoproteasome assembly
Source: J Cell Sci. 2025 May 23;138(10):jcs263887. doi: 10.1242/jcs.263887 (PMC12148024; doi:10.1242/jcs.263887)
Supplement: Supplementary file 1 [file joces-138-263887_review_history.pdf]

Journal of Cell Science

PI31 is a positive regulator of 20S immunoproteasome assembly

Jason Wang, Abbey Kjellgren and George N. DeMartino
DOI: 10.1242/jcs.263887

Editor: Pedro Carvalho

Review timeline

Original submission:	29 January 2025
Editorial decision:	4 March 2025
First revision received:	23 April 2025
Accepted:	24 April 2025

Original submission

First decision letter

MS TITLE: PI31 is a positive regulator of 20S immunoproteasome assembly
AUTHORS: Jason Wang; Abbey Kjellgren; George N. DeMartino
ARTICLE TYPE: Research Article

Dear Dr DeMartino,

We have now reached a decision on the above manuscript.

To see the reviewers' reports and a copy of this decision letter, please go to:

As you will see, the reviewers gave favourable reports but raised some critical points that will require amendments to your manuscript. I hope that you will be able to carry these out because I would like to be able to accept your paper, depending on further comments from reviewers.

Reviewer 1

Advance summary and potential significance to field

In this study, Wang et al. demonstrated that PI31, previously identified as a proteasome inhibitor, plays a role in the molecular assembly of the immunoproteasome induced by IFN-γ. PI31 deficiency did not affect the assembly of the constitutive proteasome but led to the accumulation of pro-peptide of β subunits and the proteasome assembly chaperone POMP, resulting in decreased proteasome activity and impaired immunoproteasome assembly under IFN treatment.
This study reveals that PI31 is crucial for immunoproteasome function rather than the constitutive proteasome, as previously thought, and suggests an immunoproteasome-specific assembly mechanism. These findings contribute to a deeper understanding of proteasome function in various pathological conditions and have significant implications for understanding and developing therapeutic strategies for diseases caused by immunoproteasome dysfunction, such as autoinflammatory disorders.
The experiments were logically designed and well conducted. The results are clear and highly reliable. Given the broad readership of the Journal of Cell Science, the publication of this manuscript in the journal is of great significance. However, to further clarify the importance of the authors' claims and findings, additional experiments and responses

© 2025. Published by The Company of Biologists under the terms of the Creative Commons Attribution License (https://creativecommons.org/licenses/by/4.0/).

would be beneficial. I believe that the acceptance of this manuscript would require the inclusion of additional experimental results addressing the major comments.

Comments for the author

Major comments:

1. It would be helpful to clarify how PI31 is responsible for the immunoproteasome assembly. To address this, the authors may consider examining whether immunoproteasome assembly is restored by reintroducing a PI31 expression construct into PI31 KO cells. If the add-back experiment successfully restores assembly, it would be informative to further assess whether the reintroduction of a C-terminally truncated form of PI31, which is thought to lose its association with the proteasome, has the same effect.

2. Further clarification on the selective role of PI31 in immunoproteasome assembly would strengthen the conclusions. To this end, it would be of help to investigate whether PI31 associates with specific ß subunits or assembly chaperones by immunoprecipitation of PI31 or ß subunits. Demonstrating any differences in the association of PI31 with ß subunits in the presence or absence of IFNγ treatment would provide stronger support for the conclusions. Overexpression of PI31 followed by immunoprecipitation, native PAGE, density gradient analysis, or similar analysis could be useful to examine its interaction with ß subunits. If, as suggested in the discussion, PI31 undergoes proteasomal degradation similar to POMP, its association with the proteasome under proteasome inhibitor treatment could be explored.

3. To evaluate the impact of reduced immunoproteasome assembly in PI31 KO cells on intracellular proteostasis, it may be informative to perform western blot analysis for ubiquitin and assess the accumulation of IFN-γ-induced oxidized proteins, as reported by Seifert et al. (Cell, 2010).

4. In the section on page 9 referring to Fig. 2, it would be beneficial to discuss the accumulation of ß subunit pro-peptide. Specifically, the authors should mention the decrease in mature forms of ß1i, ß2i, and ß5i, as well as the increased levels of pro-peptides in IFN-γ-treated PI31 KO cells. Also, it would be helpful to provide an explanation for the increase in pro-peptides of the constitutive subunits ß2c and ß5c in PI31 KO cells under IFN-γ treatment.

5. To confirm that PI31 KO cells respond appropriately to IFNγ signaling, it may be useful to verify the expression levels of ßi subunits and general IFN-γ-responsive genes. Since the precise cause of defective immunoproteasome assembly remains unclear, confirming the expression levels of all 20S subunits by qPCR or RNA-Seq could help exclude the possibility that the defect arises from decreased expression of proteasome subunits.

6. It would be appreciated if the authors could provide the raw image of western blotting data presented in Supplementary Figure 5.

7. Regarding Figure 4A, it would be helpful to include the results of activity staining with HAP1 in HepG2 cells, in addition to the Ac-PAL-amc assay. Furthermore, including western blotting results for PA28 in HepG2 cells would provide additional clarity.

Minor comments:
1. There appears to be a garbled section in the citation on line 6 of page 10. It needs to be corrected.

© 2025. Published by The Company of Biologists under the terms of the Creative Commons Attribution License (https://creativecommons.org/licenses/by/4.0/).

Reviewer 2

Advance summary and potential significance to field

PI31 is generally regarded as an inhibitor of the mature 20S proteasome (it was first identified as such by the DeMartino lab), particularly after cryo-EM studies showed it to bind within the 20S complex in a way that impairs access to the protease active sites without getting degraded. Previous work from the DeMartino lab demonstrated that PI31 also bound to the 20Si immunoproteasome but in a way that led to its gradual degradation, yielding a more modest effect on proteolytic activity.

Key Results
Wang et al. now provide several lines of evidence that PI31 promotes 20Si (immunoproteasome) assembly. Native PAGE gels and Western blotting data of IFNy treated PI31 KO cells suggests that PI31 is involved in a late stage of 20Si assembly, likely involving either insertion of appropriate ï□¢ subunits leading to the 15S intermediate, the fusion of two half proteasomes, or both. They also find that most of the reduction of proteasome activity in IFNy-treated PI31 KO cells is caused by a substantial reduction in the abundance and activity of 20Si-PA28 holoenzyme. This effect is caused by an apparent shortage of 20Si, and not PA28.

Validity
In general, the interpretation of data and conclusions drawn by the authors are sound.

Significance
While in vitro data has shown PI31 to be in inhibitor of 20Sc, the true biological function(s) of PI31 has largely remained a mystery. Work on the yeast ortholog of PI31, called Fub1, indicated loss of Fub1 caused sensitivity to proteotoxic stress with little change in 20S subunit levels and at best a minor increase in peptidase activity (yeast do not have a 20Si). The results from Wang et al. is a substantial advance in the understanding of PI31's biological function in vivo, although dual roles in proteasome assembly (direct or indirect) and mature proteasome inhibition, at least for 20Si, are not mutually exclusive.

Data and Methodology
The figures and data are well organized and presented clearly.

Analytical Approach
The data analysis and statistical tests performed appear straightforward and valid.

Comments for the author

Major points:
No major points of concern. The exact mechanism by which PI31 increases 20Si assembly remains unknown, but this study makes it clear that this is actually a question that needs to be addressed. It would have been nice to see PI31 complexed with an assembly intermediate(s), but the reasons for not seeing this could be technical and difficult to overcome. It remains possible, as the authors concede, that PI31 acts to enhance 20Si assembly indirectly. The authors were careful not to overstate their conclusions, so I am fine with this.

Minor points:

Please capitalize Western throughout the text when referring to the method.

© 2025. Published by The Company of Biologists under the terms of the Creative Commons Attribution License (https://creativecommons.org/licenses/by/4.0/).

The scaling on some of the proteasome activity bar graphs is a little odd. See Fig 3, Sup Fig 4B, and Sup Fig 6B Ac-ANW-amc. Not sure if this was intentional but wanted to point it out if it was not.

In Fig 5B, is it possible to make it more obvious which sets of blots are done with and without IFNy treatment?

Page 10, Line 15, something is strange with the 2014 reference.
Page 16, Lines 55-58, "component of" duplicated.

Clarity and Context
The text was written with clarity and context.

References
The authors reference appropriate literature.

Reviewer 3

Advance summary and potential significance to field

This study shows the the factor known as PI31 has a major impact on proteasome assembly, with the twist that this effect operates only on the immunoproteasome or rather the 20S component of the immunoproteasome (20Si), and not on the more commonly expressed constitutive 20S (20Sc). The paper is clean and complete, with careful biochemical analysis such as is increasingly rare to see these days unfortunately. Overall the argument is very convincing. Actually when something is a major proteasome assembly factor it is usually plain to see with the right experiments, and this is no exception.

Is it interesting? Yes, absolutely, and for a number of reasons. PI31 is a unique and remarkable regulator of the proteasome, and this study strongly adds to its fascination and mystery. As the authors recount, many functions have been attributed to PI31, and it is largely unclear how it accomplishes them, with the exception of proteasome inhibition, its "original" function, where recent structural work has been clarifying. PI31 is a powerful proteasome inhibitor, and as intuitive as it may seem for a proteasome inhibitor to function physiologically mainly as a proteasome inhibitor, it remains to be seen where this is the case, and indeed the present work does not provide support to that idea. Indeed, it is up in the air whether the key physiological function of PI31 is the same in different organisms (such as yeast and mammals, in that yeast does not have an immunoproteasome or anything in that vein), and whether PI31 even has a single key function that would explain all of the phenomona that have been associated with it genetically.

The paper leaves a paradox in that this assembly factor is not or has not been found as a component of any of the (many) assembly complexes of the 20Si. (To date all recognized perotesome assembly factors have been found on assembly intermediates.) While in some sense a weakness this is also an interesting problem and will invite additional analysis. Perhaps it actually is a component, but because of instability at some level such an intermediate is hard to document. In any case, due diligence was done and this does not affect the suitability of this study for publication in my view.

Comments for the author

I think the data are good, and I have only very minor comments on the text.

© 2025. Published by The Company of Biologists under the terms of the Creative Commons Attribution License (https://creativecommons.org/licenses/by/4.0/).

page 4 line 30: I wonder, of all papers to cite, is it best to cite the Marshall and Viersta work, since multiple papers from them on this topic have been retracted.
page 7 line 30: typo
page 9 line 21: If an effect does not reach statistical significance I think it should not be referred to as an effect but at best an apparent effect or something like that. It's not an effect at all unless it passes this test.
Page 10 line 16: typo

First revision

Author response to reviewers' comments

AUTHORS' RESPONSE TO REVIEWER COMMENTS

Author Response:

We thank the reviewers for thoughtful and knowledgeable evaluation of our manuscript. The manuscript has been revised in response to reviewer comments. The revisions include the addition and explanation of new experimental data, editing of the text to improve clarity and provide fuller explanations of previous data, and correction of a number of both noted and unnoted typographical errors. We have also replaced the HAP1 actin segment of Figure 1 D that was incorrectly repeated from Figure S6. The correct blot has been substituted. None of these changes affects any conclusions of the original manuscript. We believe that these revisions have improved the manuscript significantly. Point-by-point responses to specific reviewer comments are given below and are presented in bold typeface.

Reviewer 1

In this study, Wang et al. demonstrated that PI31, previously identified as a proteasome inhibitor, plays a role in the molecular assembly of the immunoproteasome induced by IFN-γ. PI31 deficiency did not affect the assembly of the constitutive proteasome but led to the accumulation of pro-peptide of ß subunits and the proteasome assembly chaperone POMP, resulting in decreased proteasome activity and impaired immunoproteasome assembly under IFN treatment.
This study reveals that PI31 is crucial for immunoproteasome function rather than the constitutive proteasome, as previously thought, and suggests an immunoproteasome-specific assembly mechanism. These findings contribute to a deeper understanding of proteasome function in various pathological conditions and have significant implications for understanding and developing therapeutic strategies for diseases caused by immunoproteasome dysfunction, such as autoinflammatory disorders.
The experiments were logically designed and well conducted. The results are clear and highly reliable. Given the broad readership of the Journal of Cell Science, the publication of this manuscript in the journal is of great significance. However, to further clarify the importance of the authors' claims and findings, additional experiments and responses would be beneficial. I believe that the acceptance of this manuscript would require the inclusion of additional experimental results addressing the major comments.

Author Response:
We thank the reviewer for his/her overall enthusiasm for the work.
1. It would be helpful to clarify how PI31 is responsible for the immunoproteasome assembly. To address this, the authors may consider examining whether immunoproteasome assembly is restored by reintroducing a PI31 expression construct into PI31 KO cells. If the add-back experiment successfully restores assembly, it would be informative to further assess whether the reintroduction of a C- terminally truncated form of PI31, which is thought to lose its association with the proteasome, has the same effect.

© 2025. Published by The Company of Biologists under the terms of the Creative Commons Attribution License (https://creativecommons.org/licenses/by/4.0/).

Author Response:

The reviewer correctly notes that a rescue experiment in which exogenous expression of PI31 in PI31 KO cells restores interferon-induced immunoproteasome content and activity to normal levels would provide additional support for a role of PI31 as a 20Si assembly factor. We have attempted multiple versions of this experiment. However, a meaningful interpretation of the results of these experiments is confounded by several features of PI31 action. Any rescue experiment will require exogenous expression of PI31 at a level that closely matches that of endogenous PI31 in wild-type cells. Unfortunately, we have been unable to tune PI31 expression to meet this requirement. As shown in the current work and in previously published work by others (Zaiss *et al*, 2002), high cellular levels of PI31 also inhibit interferon-induced immunoproteasome content and activity, possibly because of a scaffold-like action of PI31. Our current and previous work show that high levels of PI31 also inhibit the activity of constitutive proteasomes, possibly by direct inhibition of fully assembled proteasome (Wang *et al*, 2024). The latter effect may reflect a cellular activity that matches the *in vitro* activity of PI31. Thus, future work will be required to develop a cell system where PI31 expression can be precisely tuned to match normal endogenous levels. We have added text to the Discussion to address and explain this important issue and to note the limitations of our current data.

2. Further clarification on the selective role of PI31 in immunoproteasome assembly would strengthen the conclusions. To this end, it would be of help to investigate whether PI31 associates with specific β subunits or assembly chaperones by immunoprecipitation of PI31 or β subunits. Demonstrating any differences in the association of PI31 with β subunits in the presence or absence of IFNγ treatment would provide stronger support for the conclusions. Overexpression of PI31 followed by immunoprecipitation, native PAGE, density gradient analysis, or similar analysis could be useful to examine its interaction with β subunits. If, as suggested in the discussion, PI31 undergoes proteasomal degradation similar to POMP, its association with the proteasome under proteasome inhibitor treatment could be explored.

Author Response:

The reviewer proposes an immunoprecipitation experiment with the goal of detecting interactions between PI31 and specific □i subunits. Indeed, we have attempted multiple variants of this approach, none of which has been reproducibly successful in documenting an interaction between PI31 and proteasome subunits during interferon treatment. We have not included these negative data as figures in the manuscript but have noted these unsuccessful efforts in revised text. These results are consistent with the negative results of other experiments designed to detect PI31 interactions with assembly intermediates. A discussion of possible reasons for failure to identify PI31 as a component of assembly intermediates was presented in the original manuscript and is retained in the revised manuscript. This noted limitation of our work and has been commented on by other reviewers as appropriate.

3. To evaluate the impact of reduced immunoproteasome assembly in PI31 KO cells on intracellular proteostasis, it may be informative to perform western blot analysis for ubiquitin and assess the accumulation of IFN-γ-induced oxidized proteins, as reported by Seifert et al. (Cell, 2010).

Author Response:

The reviewer suggests an experiment to measure possible effects of altered immunoproteasome function in PI31 KO cells based on a previous report (Seifert *et al*, 2010). However, we note that the results of that study involving differences of constitutive and immuno- proteasomes on ubiquitylated proteins, has been questioned by others (Nathan *et al*, 2013). In fact, we also have been unable to detect differences in the steady state levels of polyubiquitylated proteins in PI31 wild-type and KO cells with or without interferon treatment. A typical result is shown here for reviewer inspection.

Note:We have removed unpublished data that had been provided for the referees in confidence.

4. In the section on page 9 referring to Fig. 2, it would be beneficial to discuss the accumulation of β subunit pro-peptide. Specifically, the authors should mention the decrease in

© 2025. Published by The Company of Biologists under the terms of the Creative Commons Attribution License (https://creativecommons.org/licenses/by/4.0/).

mature forms of β1i, β2i, and β5i, as well as the increased levels of pro-peptides in IFN-γ-treated PI31 KO cells. Also, it would be helpful to provide an explanation for the increase in pro-peptides of the constitutive subunits β2c and β5c in PI31 KO cells under IFN-γ treatment.

<u>Author Response</u>:
As requested, we have noted the accumulation of propeptides for □i subunits in PI31 KO cells shown in Figure 2. This effect is the subject of further analysis in experiments shown in Figure 4, Figure 6, Figure S4, and Figure S5. The reviewer also correctly notes that the images presented in Figure 2A show modestly enhanced levels of propeptides for □2c and □5c subunits. Unlike the effects on □i subunits, this finding was not reproducible among multiple independent experiments (see other noted figures). Moreover, it is not a feature in HepG2 cells. Therefore, we have elected not to comment on this issue.

5. To confirm that PI31 KO cells respond appropriately to IFNγ signaling, it may be useful to verify the expression levels of βi subunits and general IFN-γ-responsive genes. Since the precise cause of defective immunoproteasome assembly remains unclear, confirming the expression levels of all 20S subunits by qPCR or RNA-Seq could help exclude the possibility that the defect arises from decreased expression of proteasome subunits.

<u>Author Response</u>:
The reviewer raises an important point about excluding possible effects of PI31 on □i subunit transcription. We have performed real time qPCR as suggested and show that PI31 KO cells have no defect in expression of mRNAs of immunoproteasome subunits. We have presented these data in Supplemental Figure 9 in the revised manuscript.

6. It would be appreciated if the authors could provide the raw image of western blotting data presented in Supplementary Figure 5.

<u>Author Response</u>:
We have provided representative Western blotting images as requested in new Supplemental Figure 7E.

7. Regarding Figure 4A, it would be helpful to include the results of activity staining with HAP1 in HepG2 cells, in addition to the Ac-PAL-amc assay. Furthermore, including western blotting results for PA28 in HepG2 cells would provide additional clarity.

<u>Author Response</u>:
We have provided activity staining data as indicated and western blotting results for PA28 as requested. Because these data are from a different independent experiment from the data shown in Figure 4, we have provided them in a separate figure (Supplemental Figure 6 in the revised manuscript). We have added other panels from that same experiments for the purpose of comparison.

Minor comments:
1. There appears to be a garbled section in the citation on line 6 of page 10. It needs to be corrected.

<u>Author Response</u>:
We have corrected the noted word processing error and apologize for this oversight.

Reviewer 2: SUMMARY OF THE ADVANCE MADE IN THIS PAPER AND ITS POTENTIAL SIGNIFICANCE TO THE FIELD

PI31 is generally regarded as an inhibitor of the mature 20S proteasome (it was first identified as such by the DeMartino lab), particularly after cryo-EM studies showed it to bind within the 20S complex in a way that impairs access to the protease active sites without getting degraded.

© 2025. Published by The Company of Biologists under the terms of the Creative Commons Attribution License (https://creativecommons.org/licenses/by/4.0/).

Previous work from the DeMartino lab demonstrated that PI31 also bound to the 20Si immunoproteasome but in a way that led to its gradual degradation, yielding a more modest effect on proteolytic activity.

Key Results
Wang et al. now provide several lines of evidence that PI31 promotes 20Si (immunoproteasome) assembly. Native PAGE gels and Western blotting data of IFNγ treated PI31 KO cells suggests that PI31 is involved in a late stage of 20Si assembly, likely involving either insertion of appropriate ▫ subunits leading to the 15S intermediate, the fusion of two half proteasomes, or both. They also find that most of the reduction of proteasome activity in IFNγ-treated PI31 KO cells is caused by a substantial reduction in the abundance and activity of 20Si-PA28 holoenzyme. This effect is caused by an apparent shortage of 20Si, and not PA28.

Validity
In general, the interpretation of data and conclusions drawn by the authors are sound.

Significance
While in vitro data has shown PI31 to be in inhibitor of 20Sc, the true biological function(s) of PI31 has largely remained a mystery. Work on the yeast ortholog of PI31, called Fub1, indicated loss of Fub1 caused sensitivity to proteotoxic stress with little change in 20S subunit levels and at best a minor increase in peptidase activity (yeast do not have a 20Si). The results from Wang et al. is a substantial advance in the understanding of PI31's biological function in vivo, although dual roles in proteasome assembly (direct or indirect) and mature proteasome inhibition, at least for 20Si, are not mutually exclusive.

Data and Methodology
The figures and data are well organized and presented clearly.

Analytical Approach
The data analysis and statistical tests performed appear straightforward and valid.

<u>Author Response</u>:
We thank the reviewer for the overall positive comments about the manuscript.

SUGGESTIONS TO AUTHORS

Major points:
No major points of concern. The exact mechanism by which PI31 increases 20Si assembly remains unknown, but this study makes it clear that this is actually a question that needs to be addressed. It would have been nice to see PI31 complexed with an assembly intermediate(s), but the reasons for not seeing this could be technical and difficult to overcome. It remains possible, as the authors concede, that PI31 acts to enhance 20Si assembly indirectly. The authors were careful not to overstate their conclusions, so I am fine with this.

<u>Author Response</u>:
We thank the reviewer these comments.

Minor points:

Please capitalize Western throughout the text when referring to the method.

<u>Author Response</u>:
We have capitalized “Western” throughout.

The scaling on some of the proteasome activity bar graphs is a little odd. See Fig 3, Sup Fig 4B, and Sup Fig 6B Ac-ANW-amc. Not sure if this was intentional but wanted to point it out if it was not.

© 2025. Published by The Company of Biologists under the terms of the Creative Commons Attribution License (https://creativecommons.org/licenses/by/4.0/).

Author Response:
We intended the scaling as shown to include "100" as a control value to allow easy comparison of other values. Scaling at intervals of 100 for other data makes the y axis overly dense, in our opinion. We would prefer to retain figures in their current state, but will alter them if the reviewer or editor deem it desirable.

In Fig 5B, is it possible to make it more obvious which sets of blots are done with and without IFNy treatment?

Author Response:
We have added notations on the figure to better indicate which data are associated with interferon treatment.

Page 10, Line 15, something is strange with the 2014 reference.

Author Response:
As noted above, we have corrected the word processing error, and apologize for this oversight.

Page 16, Lines 55-58, "component of" duplicated.

Author Response:
The duplicated word has been removed.

Reviewer 3: SUMMARY OF THE ADVANCE MADE IN THIS PAPER AND ITS POTENTIAL SIGNIFICANCE TO THE FIELD

This study shows the the factor known as PI31 has a major impact on proteasome assembly, with the twist that this effect operates only on the immunoproteasome or rather the 20S component of the immunoproteasome (20Si), and not on the more commonly expressed constitutive 20S (20Sc). The paper is clean and complete, with careful biochemical analysis such as is increasingly rare to see these days unfortunately. Overall the argument is very convincing. Actually when something is a major proteasome assembly factor it is usually plain to see with the right experiments, and this is no exception. Is it interesting? Yes, absolutely, and for a number of reasons. PI31 is a unique and remarkable regulator of the proteasome, and this study strongly adds to its fascination and mystery. As the authors recount, many functions have been attributed to PI31, and it is largely unclear how it accomplishes them, with the exception of proteasome inhibition, its "original" function, where recent structural work has been clarifying. PI31 is a powerful proteasome inhibitor, and as intuitive as it may seem for a proteasome inhibitor to function physiologically mainly as a proteasome inhibitor, it remains to be seen where this is the case, and indeed the present work does not provide support to that idea. Indeed, it is up in the air whether the key physiological function of PI31 is the same in different organisms (such as yeast and mammals, in that yeast does not have an immunoproteasome or anything in that vein), and whether PI31 even has a single key function that would explain all of the phenomona that have been associated with it genetically.
The paper leaves a paradox in that this assembly factor is not or has not been found as a component of any of the (many) assembly complexes of the 20Si. (To date all recognized perotesome assembly factors have been found on assembly intermediates.) While in some sense a weakness this is also an interesting problem and will invite additional analysis. Perhaps it actually is a component, but because of instability at some level such an intermediate is hard to document. In any case, due diligence was done and this does not affect the suitability of this study for publication in my view.

Author Response:
We thank the reviewer for the overall positive comments about the manuscript.

© 2025. Published by The Company of Biologists under the terms of the Creative Commons Attribution License (https://creativecommons.org/licenses/by/4.0/).

SUGGESTIONS TO AUTHORS

I think the data are good, and I have only very minor comments on the text.
page 4 line 30: I wonder, of all papers to cite, is it best to cite the Marshall and Viersta work, since multiple papers from them on this topic have been retracted.

Author Response:
We concur that inclusion of the Marshall reference was ill-advised. We have removed and replaced with another reference on a similar topic.

page 7 line 30: typo

Author Response:
We have corrected the noted word processing error.

page 9 line 21: If an effect does not reach statistical significance I think it should not be referred to as an effect but at best an apparent effect or something like that. It's not an effect at all unless it passes this test.

Author Response:
We have reworded the text as suggested.

Page 10 line 16: typo

Author Response:
We have corrected the indicated word processing error, as noted above.

CITED REFERENCES
Nathan JA, Spinnenhirn V, Schmidtke G, Basler M, Groettrup M, Goldberg AL (2013) Immuno- and constitutive proteasomes do not differ in their abilities to degrade ubiquitinated proteins. *Cell* 152: 1184- 1194

Seifert U, Bialy LP, Ebstein F, Bech-Otschir D, Voigt A, Schroter F, Prozorovski T, Lange N, Steffen J, Rieger M *et al* (2010) Immunoproteasomes preserve protein homeostasis upon interferon-induced oxidative stress. *Cell* 142: 613-624

Wang J, Kjellgren A, and , DeMartino GN (2024) Differential interactions of the proteasome inhibitor PI31 with constitutive and immuno-20S proteasomes. *Biochemistry* 63: 1000-1015

Zaiss DM, Standera S, Kloetzel PM, Sijts AJ (2002) PI31 is a modulator of proteasome formation and antigen processing. *Proc Natl Acad Sci U S A* 99: 14344-14349

Second decision letter

MS ID#: jcs.263887R1

MS Title: PI31 is a positive regulator of 20S immunoproteasome assembly
Authors: Jason Wang; Abbey Kjellgren; George N. DeMartino
Article Type: Research Article

Dear Dr DeMartino,

I am happy to tell you that your manuscript has been accepted for publication in Journal of Cell Science, pending standard publication integrity checks.

© 2025. Published by The Company of Biologists under the terms of the Creative Commons Attribution License (https://creativecommons.org/licenses/by/4.0/).
